# Biofabrication, statistical optimization, and characterization of collagen nanoparticles synthesized via *Streptomyces* cell-free system for cancer therapy

**DOI:** 10.1038/s41598-025-29189-7

**Published:** 2025-12-13

**Authors:** Noura El-Ahmady El-Naggar, Shaimaa Elyamny, Ahmad G. Shitifa, Asmaa Atallah El-Sawah

**Affiliations:** 1 Department of Bioprocess Development, Genetic Engineering and Biotechnology Research Institute, City of Scientific Research and Technological Applications (SRTA-City), New Borg El-Arab City, Alexandria 21934 Egypt; 2 Electronic Materials Research Department, Advanced Technology and New Materials Research Institute, City of Scientific Research and Technological Applications (SRTA-City), P.O. Box 21934, New Borg El Arab City, Alexandria Egypt; 3 Chemistry Department, Faculty of Science, Mansoura University, Mansoura, Egypt; 4 Clinical Pathology Department, Official Mansoura University Hospital, Mansoura, 35516 Egypt

**Keywords:** Collagen nanoparticles, *Streptomyces viridochromogenes*, Biofabrication, Central composite rotatable design, Artificial neural networks, Characterization, Antioxidant, HepG2 cancer cell lines, Ehrlich ascites carcinoma, Biochemistry, Biological techniques, Biotechnology, Chemistry, Drug discovery, Nanoscience and technology

## Abstract

Collagen nanoparticles (CollNPs) exhibit low antigenicity, high surface area, non-toxicity, and excellent biocompatibility, making them promising for diverse biomedical applications. This study reports, for the first time, an eco-friendly, sustainable microbial-mediated biosynthesis of CollNPs using the cell-free system of *Streptomyces viridochromogenes*. UV–Vis spectroscopy showed a distinct absorption peak at 240 nm. A synergistic modeling approach combining Central Composite Rotatable Design (CCRD) and Artificial Neural Networks (ANN) were used to optimize biosynthesis parameters. The ANN identified the predicted optimal conditions at pH 7, collagen concentration 20 mg/mL, incubation time 115 h, and temperature 32 °C, with a theoretical maximum yield of 19.87 mg/mL. Experimental validation confirmed the model’s accuracy, yielding 19.1 mg/mL Sv-CollNPs, closely matched the theoretical prediction ANN exhibited higher predictive accuracy than CCRD, confirming its robustness for process optimization. TEM revealed hollow, spherical Sv-CollNPs with an average diameter of 30.41 nm. FTIR analysis identified NH, CH, C = O, and OH functional groups contributing to nanoparticles formation and stabilization. The ζ-potential was − 19.4 mV, indicating good colloidal stability. The Sv-CollNPs displayed strong antioxidant and anti-hemolytic activities, alongside notable anticancer activity . The IC_50_ values were 7.94 ± 0.6 µg/mL (MCF-7), 13.89 ± 1.0 µg/mL (HepG2), and 22.06 ± 1.6 µg/mL (HCT116). *In vivo* experiments were conducted on six groups (*n* = 8 per group). Treatment with native collagen, Sv-CollNPs, and DOX reduced EAC tumor growth by  65.66%, 83.84%, and 85.86%, respectively. The combined treatment of Sv-CollNPs and DOX achieved the highest tumor inhibition (97.98%), exceeding the effects of the individual treatments. In conclusion, this study introduces *S. viridochromogenes* as a novel microbial source for collagen nanoparticles biosynthesis and demonstrates the superior predictive performance of ANN-based modeling. The findings reveal that biosynthesized Sv-CollNPs, especially when combined with DOX, exhibit strong therapeutic potential and represent an eco-friendly, innovative strategy for anticancer nanomedicine development.

## Introduction

Over the past few decades, nanoparticles (NPs) have been developed and extensively investigated for various biomedical applications due to their remarkable physicochemical properties. Their nanoscale dimensions, large surface area-to-volume ratio, and tunable surface functionality enable them to achieve site-specific drug delivery, enhance therapeutic efficacy, and minimize systemic toxicity. Based on their composition and structural nature, NPs are generally classified into metallic, semiconductor, polymeric, carbon-based, and oxide or ceramic nanoparticles. Metallic nanoparticles such as cobalt (Co), palladium (Pd), silver (Ag), and gold (Au) along with their corresponding oxides possess unique optical, magnetic, and catalytic properties that make them vital for diverse technological and biomedical applications in drug delivery systems, diagnostic imaging, biosensing, and cancer therapy^1,2^. While metallic NPs hold great promise in technological and biomedical fields, their uncontrolled release and persistence in the environment demand rigorous assessment of their long-term ecological and health impacts. The accumulation of non-degradable metallic nanoparticles in agricultural systems poses a significant threat to agricultural sustainability and food security, ultimately impacting human health. Numerous studies have reported that excessive accumulation of metallic NPs in the body can lead to severe adverse effects, including neurotoxicity, nephrotoxicity, and immunogenic responses, primarily resulting from their deposition in vital organs such as the liver^3,4^. Additionally, polymeric nanoparticles possess several advantageous properties such as nanoscale size, high surface area-to-volume ratio, facile structural and surface modification, excellent biocompatibility, biodegradability and capacity to encapsulate a wide range of therapeutic molecules. These attributes make them highly suitable for the efficient delivery of anticancer agents^5,6^, in wound healing applications.

Collagen is a naturally occurring fibrous protein that constitutes approximately 25% of total proteins in humans and is vital for the structural integrity of tissues in both vertebrates and many other multicellular organisms. It possesses several favorable properties, including sustainability, structural stability, porosity, low immunogenicity, biocompatibility, and biodegradability, making it a valuable biomaterial for biomedical applications^7^. Traditionally, collagen is extracted from various animal tissues such as horse tendons, frog and sheep skin, rat tails, chicken, porcine skin, duck legs, bovine skin and tendons, intestines, and bladder mucosa of pigs. However, the characteristics of collagen vary depending on the source species and tissue type. The use of animal-derived collagen may be limited by risks of allergic reactions and pathogen transmission^8,9^. In this regard, growing attention has been directed toward identifying safer and more sustainable sources of collagen. Marine organisms including fish, jellyfish, and sponges have recently emerged as promising alternative sources to traditional mammalian-derived collagen^10^. Marine collagen offers several advantages over mammalian collagen, including ease of extraction, lower production cost, reduced immunogenicity, non-cytotoxicity, and improved physicochemical stability. Moreover, its non-mammalian origin contributes to greater cultural and religious acceptability, making it particularly suitable for a wide range of biomedical and pharmaceutical applications^11,12^. Clinical studies have demonstrated its anti-aging effects, ability to enhance skin elasticity, reduce wrinkles, accelerate wound healing, and even promote bone regeneration in animal models^10^. The majority of current researches have remained centered on collagen extraction and its fundamental biomedical applications, with relatively limited attention given to the synthesis of collagen-based nanostructures via eco-friendly, microbially mediated approaches.

Collagen nanoparticles (CollNPs) have attracted increasing attention due to their nanoscale dimensions and enhanced functionalities. CollNPs combine the intrinsic biocompatibility and biodegradability of native collagen with unique nanostructural advantages such as large surface area, low toxicity, high cationic charge density, and superior bioactivity. These properties render them highly suitable for controlled drug delivery, bone and tissue regeneration, and wound healing application^13–15^. Furthermore, CollNPs exhibit improved solubility, stability, prolonged half-life, and drug-release control compared with bulk collagen. Their stimuli-responsive behavior and tunable scaffold properties make them suitable for tissue engineering and regenerative medicine^13^. CollNPs have also been explored for their potential in nerve regeneration. For instance, Zhang et al.^16^ demonstrated their effectiveness in facial nerve repair. Their utility as drug delivery vehicles for cancer therapeutics has also been established. Incorporation of CollNPs into 3D bioprinting scaffolds has significantly reduced the need for donor cartilage transplantation and other invasive interventions^17^. Moreover, the encapsulation of bioactive compounds, such as silymarin, into CollNPs has shown improved neuroprotective effects and enhanced bioavailability at minimal doses against ischemia/reperfusion injury models by enhancing its bioavailability in the bloodstream and targeting^18^.

Various chemical and physical methods have been employed for the synthesis of CollNPs^17^. However, many conventional synthesis routes involve the use of toxic solvents and harsh conditions, posing risks to the environment and human health. Therefore, there is a growing need to develop eco-friendly, biogenic methods that utilize safe microbial systems capable of synthesizing collagen nanoparticles in a sustainable way. In this context, green synthesis techniques utilizing biological systems such as plants, algae, yeast, fungi, bacteria, and actinomycetes have emerged as a safer, more cost-effective, and environmentally sustainable alternative^19^. However, the microbial synthesis of CollNPs, particularly using actinomycetes remains largely unexplored and insufficiently optimized. This research gap highlights the need for systematic process optimization and predictive modeling to ensure efficient, high-yield, and reproducible biosynthesis.

Recent innovations in computational prediction technologies are revolutionizing biofabrication. Traditionally, production of bio-based nanomaterials largely depended on repetitive experimental trials, a method that was time-consuming, costly, and inefficient. The integration of computational modeling with machine learning now makes it possible to simulate complex biological systems and predict the most effective synthesis conditions before laboratory testing begins. Among these tools, artificial neural networks (ANNs) have gained considerable attention for their ability to analyze large, noisy datasets with high precision in bioprocesses such as ncanoparticles biosynthesis due to their powerful modeling and predictive capabilities. ANNs can generate precise, efficient predictive models that enhance reproducibility and scalability while reducing material consumption, energy use, and experimental waste. In nanoparticles synthesis, parameters including substrate concentration, pH, temperature, reaction time, and type of reducing agent directly influence particle size, shape, stability, and yield. This hybrid approach not only improves predictive accuracy but also promotes sustainability by minimizing experimental trials and resource use. The integration of RSM with ANNs offers a synergistic modeling approach that combines the strengths of statistical and artificial intelligence tools to develop accurate predictive models^20^. This combination not only improves predictive accuracy but also contributes to sustainability by minimizing raw material use, energy consumption, and experimental waste. Recent studies have shown that ANN outperforms traditional RSM/CCD in predictive accuracy for nanoparticle biosynthesis and enables reduction of experimental runs and material waste^21–23^.

 This study aimed to develop an eco-friendly strategy for the biosynthesis of ultrafine CollNPs using a newly isolated strain, *Streptomyces* sp. NEAA-5, as a potential producer of Sv-CollNPs. Additionally, the study aimed to optimize the key parameters for maximal nanoparticles yield using CCRD. The biosynthetic process was subsequently validated and predicted through ANN-based analysis of data obtained from the CCRD. The resultant Sv-CollNPs were comprehensively characterized to determine their physicochemical properties. Their antioxidant activity, *in vitro* cytotoxicity against cancer cell lines, and *in vivo* anticancer potential were also evaluated. In addition, detailed identification of *Streptomyces* sp. NEAA-5 was performed using cultural, morphological, physiological, and genetic techniques.

## Materials and methods

### Cultural conditions and cell-free supernatant (CFS) preparation

A strain of *Streptomyces* sp. NEAA-5, kindly provided by the first author. The strain was cultured on Petri plates containing starch–nitrate agar medium. The preparation of the cultivation medium was initiated by formulating starch–nitrate medium, the medium’s pH was adjusted to 7.0 ± 0.2 prior to sterilization. Following pH adjustment, the medium was sterilized, poured into sterile Petri plates. The *Streptomyces* sp. NEAA-5 strain was then inoculated onto the prepared starch–nitrate agar plates and incubated at 30 °C for 7 days^24^. For long-term preservation, *Streptomyces* sp. NEAA-5 was maintained at − 20 °C in 20% (v/v) glycerol as spore suspensions. Six discs (9 mm in diameter) from a starch–nitrate agar culture of *Streptomyces* sp. NEAA-5 were transferred into Erlenmeyer flasks containing 100 mL of a medium composed of (g/L): Soluble starch, 20; MgSO_4_, 0.5; KNO_3_, 1; NaCl, 0.5; K_2_HPO_4_, 0.5; FeSO_4_.7H_2_O, 0.1 and 0.3 g yeast extract; 1 L of distilled water^25^. All chemicals were purchased from Sigma-Aldrich (St. Louis, MO, USA).The cultures were incubated for 5 days at 30 °C at 150 rpm in a rotary shaker. The mycelial biomass was then separated from the CFS through centrifugation at 4 °C for 15 min. Equal volumes of freshly prepared CFS obtained under identical growth conditions, were used for each synthesis experiment to ensure reproducibility. To further ensure experimental consistency, a large batch of CFS was initially prepared, then aliquoted into sterile containers and stored at − 20 °C to minimize batch-to-batch variation throughout the study and preserved the biochemical integrity of the supernatant throughout the entire study.

### Gas chromatography-mass spectrometry (GC-MS) analysis of CFS

The chemical compositions for fatty acids methyl esters (FAMEs) presented in the CFS were performed using a Trace GC1310-ISQ mass spectrometer (Thermo Scientific, Austin, TX, USA). Approximately 20 mg of dried CFS was subjected to transesterification using 0.5 N methanolic KOH (2.805 g KOH in 100 mL methanol). The mixture was vortexed and heated at 50 °C for 15 min, then cooled and mixed thoroughly. Five millilitres of 4 N HCl (prepared from 3.4 mL HCl in 100 mL water) was added, followed by extraction with a 1:1 mixture of petroleum ether and hexane. The upper organic layer containing FAMEs was collected and evaporated at 40 °C. The residue was redissolved in 1 mL hexane and analyzed by GC–MS using an HP-5MS silica capillary column with helium as the carrier gas. The oven temperature was programmed from 60 °C (held 2 min) to 280 °C at 8 °C/min. Fatty acid components were identified by comparison with the Wiley and NIST mass spectral libraries^26^.

### Extracellular biofabrication of Sv-CollNPs

For the biofabrication of Sv-CollNPs, 9 mL of freshly prepared CFS was mixed with 1 mL of marine collagen solution (10 mg/mL; MM Ingredients Ltd., UK). incubated for 48 h at 35 °C. The appearance of white turbidity indicated Sv-CollNPs formation. The turbidity was centrifuged at 14,000 rpm for 10 min. The resulting pellets were washed with distilled water three times to completely remove residual CFS, and then re-dispersed in distilled water. The obtained Sv-CollNPs were freeze-dried for subsequent characterization and applications.

### UV–vis spectroscopy of Sv-CollNPs

The spectroscopic analysis of Sv-CollNPs was analyzed by recording UV–vis absorbance spectra in the range of 200–800 nm using an ATI Unicam 5625 UV/VIS spectrophotometer (Vision Software, v3.20).

### CCRD and ANN models-based optimization of Sv-CollNPs biofabrication by  *Streptomyces* sp. NEAA-5

In the current study, a hybrid modeling approach using ANN and RSM was employed to optimize, validate, and predict Sv-CollNPs biosynthesis using the CFS of system of *Streptomyces* sp. NEAA-5 based on the data obtained from the central composite rotatable design (CCRD). The CCRD model was utilized to determine the optimal levels of four factors of initial pH level (X_1_), collagen conc. (X_2_), incubation period (X_3_), and temperature (X_4_) for the highest yield of Sv-CollNPs using the CFS of *Streptomyces* sp. NEAA-5. Each factor was studied using five coded values (− 2, − 1, 0, 1, and 2). The CCRD model was performed through 30 experimental runs with 3 replicates. The levels of the factors were as follow: initial pH (X_1_; 5, 6, 7, 8, and 9), collagen conc. (X_2_; 4, 8, 12, 16, and 20 mg/mL), incubation period (X_3_; 24, 48, 72, 96 and, 120 h), and temperature (X_4_; 25, 30, 35, 40 and 45 °C). The following second-order polynomial equation is used to determine the theoretical relationships between the independent factors and the efficiency of Sv-CollNPs biofabrication:

1 $$Y={\beta _0}+\sum\limits_{i} {{\beta _i}{X_i}} +\sum\limits_{{ii}} {{\beta _{ii}}{X_i}^{2}} +\sum\limits_{{ij}} {{\beta _{ij}}{X_i}{X_j}}$$

Where Y represents the predicted Sv-CollNPs biofabrication yield, and X_1_, X_2_, X_3_, and X_4_ represent the coded values of the independent factors. β₀ is the intercept term, β_i_ represents the linear coefficients, β_i__i_ represents the quadratic coefficients, and β_i_ʲ represents the interaction coefficients.

The ANN model comprised three layers. The input layer incorporated four key process variables: pH level (X_1_), collagen concentration (X_2_), incubation time (X_3_), and temperature (X_4_). These inputs were processed within a hidden layer containing neurons, functioning as the intermediary between the input and output layers. To enhance the predictive capability of the ANN model in this study, specific configuration parameters were selected. The processed data were then transmitted to a single-neuron output layer, which generated predictions for the optimal conditions required for the green synthesis of Sv-CollNPs by *Streptomyces* sp. NEAA-5.

Specific parameter settings were applied, including an NTanH (20) architecture with 5000 tours, a learning rate of 0.1, and a holdback validation strategy using a 0.2 ratio. NTanH (20) describes a hidden layer with 20 neurons activated by the hyperbolic tangent (TanH) activation function. N represents the number of neurons in the hidden layer, while TanH denotes the hyperbolic tangent activation function applied to those neurons. The selection of 20 neurons in the hidden layer was based on empirical optimization through repeated testing of different network architectures (ranging from 5 to 30 neurons). Each network architecture was evaluated using statistical performance indicators such as the mean squared error (MSE) and the coefficient of determination (R^2^). The model with 20 neurons produced the lowest MSE and highest R^2^, indicating accurate prediction.

CCRD served as the design for data-generation, whereas ANN functioned as an advanced modeling and optimization tool built upon that dataset. CCRD was initially applied as a statistical experimental approach to evaluate how the key parameters including pH, collagen concentration, incubation time, and temperature affect the production yield of Sv-CollNPs. The dataset obtained from the CCRD experiments was subsequently utilized as both training and validation input for constructing the ANN model. Model performance was assessed using the coefficient of determination (R^2^), sum of squared errors (SSE), mean absolute deviation (MAD), and root mean square error (RMSE). To verify accuracy, experimental trials were conducted to compare the predictive results from both the CCRD and ANN models with the actual Sv-CollNPs yields obtained from *Streptomyces* sp. NEAA-5.

### Characterization of Sv-CollNPs

The dimensions and morphology of the Sv-CollNPs solution were determined utilizing a TEM instrument with superior resolution (JEOL-JEM-100 CXII). The lyophilized Sv-CollNPs were gold-coated using a sputtering technique (SPI-Module). The size, shape, and morphological characteristics of the Sv-CollNPs sample were performed by SEM (JSM - model JEOL- IT200) operating at 20 kV.

Scanning electron microscopy (SEM) allows for the accessibility of EDXS, which is widely utilized to determine the composition of elements present in a material. When electrons come into contact with a material during SEM imaging, the material emits X-rays, which are picked up by the EDXS detector.

Sv-CollNPs’s surface features were examined, and the chemical components of each were identified using FTIR spectroscopy. Sv-CollNPs samples were mixed, followed by grinding with KBr pellets in order to examine the surface characteristics. The FTIR spectra of the Sv-CollNPs were obtained using a Shimadzu FTIR-8400 S spectrophotometer, which has a resolution of approximately 1 cm^− 1^ and a spectrum spanning from 4000 to 500 cm^− 1^.

Determining the crystalline properties of nanoparticles requires the use of X-ray diffractogram analysis (XRD). Bruker’s (D2 Phaser − 2nd Gen) CuKα radiation (wavelength = 1.54Å) produced at 10 mA and 30 kV operating at room temperature was used to study X-ray diffraction. The intensity of diffraction was investigated at 2º min^− 1^ scanning rate, and the measured 2θ values varied from 5º to 80º^27,28^.

The behavior of colloids or suspensions of nanoparticles can be accurately predicted using the zeta potential^29^. The Sv-CollNPs employed in this analysis were identified by phase analysis light scattering, and their zeta potential and surface charge characteristics were measured using a laser Doppler and the Malvern 3000 analytical Zetasizer software (Nano ZS, UK)^27^. The nanoparticles were diluted using deionized water, and their viscosity was adjusted to 0.8872 cP for lowering the degree of scattering effects^30^. Following the dissolution of the sample, the nanoparticles were measured at a rate of 101.9 kcps (kilo counts per second) throughout a 2 mm calibrated area for 60 s. Three separate tests were performed on the sample at a temperature of 25 °C^31^. We determined the mean hydrodynamic diameter values for the various nanoformulations.

The thermogravimetric analysis (TGA) was performed using Universal V4.5 A TA Instruments. After the Sv-CollNPs specimen had withered for an hour at 60 °C, it was carefully placed in the sample platinous pan. Next, using a thermoanalyzer model number 50-H, the samples were subjected to temperatures that ranged from room temperature to 800 °C for TGA measurements. TGA was performed in a nitrogen atmosphere at a 10 mL min^− 1^ flow rate and 10 °C per minute temperature increase. After that, the weight loss percentage was plotted versus temperature^27^. The pyrolysis pattern of Sv-CollNPs was performed by differential scanning calorimetry (DSC). The Sv-CollNPs specimen has been mounted on an aluminium sample pan after getting dried for 1 h at 60 °C. The investigation was performed in a nitrogen atmosphere with 10 °C per minute of heating rate and an average flow rate of 30 mL min^− 1^. The thermogram’s temperature varied from room temperature to 800 °C. The thermogravimetric examination findings for the first decomposition temperature of the Sv-CollNPs were used to determine the DSC upper limit. The graphical representation showed the temperature and the rate of heat flow^27^.

### Anti-oxidant assay

To assess the antioxidant activity of collagen and Sv-CollNPs, a 60 µM ABTS solution was mixed with 3 mL of MnO₂ solution (25 mg/mL). The ABTS and MnO_2_ were made in 5 mL with a concentration of 0.1 M and a pH of 7. After shaking, centrifuging, and filtering, the green-blue solution (ABTS^+^) was measured at λ 734 nm, and the absorbance (OD_control_) was adjusted to approximately 0.5. Next, mix one equal volume (1 mL) of the ABTS^+^ separately with collagen (500 µg/mL) and Sv-CollNPs (500 µg/mL) for 30 min at 37 °C and measure at 734 nm (OD_test_) using a microplate reader. The absorbance (OD_test_) was expressed as a percentage of inhibition and connected to the reduction in color intensity. The following formula is used to calculate the percentage inhibition: (OD_control_ - OD_test_)/ OD_control_ × 100; the positive control was ascorbic acid^12^.

### Anti-hemolytic assay

A blood sample from punctured rat hearts was put in a heparin tube. The erythrocytes have been isolated from the plasma after being centrifuged at 2000 rpm for 15 min. during the final wash to obtain packed erythrocyte cells. In this test protocol, hemolysis was induced by peroxyl radicals. A 200 mM solution of AAPH in phosphate-buffered saline (pH 7.4) at varying concentrations was mixed with a 10% suspension of erythrocytes prepared in PBS. After being diluted with eight volumes of PBS and mildly agitated for 2 h at 37 °C, the liquid was centrifuged for 10 min at 1500 rpm. The supernatant’s absorbance was determined at 540 nm. Eight volumes of dist. H_2_O have been combined with the mixture and treated the same way. After centrifugation, the supernatants’ absorbance (A) was measured at 540 nm along with H_2_O to evaluate if complete hemolysis occurred. The positive control that was used was L-ascorbic acid. The equation (100- (A_sample_/ A_control_ × 100%)) was used to calculate the hemolysis percentage^12^.

### *In vitro* antitumor assay

Sv-CollNPs and collagen were tested using a 3-(4, 5-Dimethylthiazol-2yl)-2, 5-diphenyl tetrazolium bromide (MTT) methodology^32^ to determine their antitumor effects against both cancer and normal cell lines. Cancer cell lines WI-38 (human lung tissue), WISH (human amnion tissue), HCT116 (human colorectal carcinoma), HeP-G2 (human hepatocellular carcinoma), and MCF-7 (human breast carcinoma) were provided by VACSERA Company (Egypt). The MTT assay was used to evaluate cytotoxicity according to the procedure of El-Sawah et al.^12^. Cytotoxicity (%) was calculated using the formula:

2 $${\text{Viability percentage }}\left( \% \right){\text{ }}={\text{ }}\left( {{{\text{A}}_{{\text{test}}}}/{\text{ }}{{\text{A}}_{{\text{control}}}}} \right){\text{ }} \times {\text{ 1}}00$$

3 $${\text{Cytotoxicity percentage }}\left( \% \right)\,=\,{\text{1}}00{\text{ }}-{\text{Viability percentage }}\left( \% \right)$$

### *In vivo* apoptosis induction assay

Adult female Swiss albino mice (8–10 weeks old, 25–30 g) were obtained from the Theodore Bilharz Research Institute (Giza, Egypt) and maintained under sterilized conditions in polyvinyl cages. Animals were supplied with a standard sterilized diet and water at a standard temperature of 26 ± 1 °C and 20% relative humidity. They were also kept on a 12-hour light/dark photoperiod. Approximately 5 × 10^5^ of Ehrlich ascites carcinoma (EAC) cells were first subcutaneously injected into each mouse for 5 days to create solid tumors with a long diameter average of ~ 0.63 ± 0.02 cm and a short diameter average of ~ 0.48 ± 0.01 cm (day 0) before beginning therapy. EAC cells were provided from Cairo University’s National Cancer Institute (NCI), Cairo, Egypt. After then, the mice were divided into six groups (*n* = 8) at random. Group I consisted of only EAC-bearing mice and was used as the control. Group II administered an injection of collagen (2 mg/kg/day) directly into the tumor; Group III administered an injection of doxorubicin (DOX) (2 mg/kg/day); Group IV administered an injection of Sv-CollNPs (2 mg/kg/day); Group V administered an injection of collagen in addition to DOX; and Group VI administered an injection of Sv-CollNPs in addition to DOX. There have been numerous prior studies using the same study design with a sample size of (*n* = 5) in each group as in El-Naggar et al.^33^ and El-Sawah et al.^34^.

Following tumor induction, mice received intraperitoneal injections of collagen, Sv-CollNPs, or DOX every 5 days (over a 20-day treatment period). According to globally recognized standards of humane endpoints, animals were euthanized if they lost more than 20% of their body weight, had ulceration or necrosis at the tumor site, were unable to get food or water, were extremely lethargic, or displayed symptoms of pain or distress like hunching, a rough coat, or difficulty breathing. Therefore, every day, the mice’s body weight, behavior, food and water intake, and general health were recorded. On day 21, mice were sacrificed under anesthesia by cervical dislocation following administration of thiopental sodium (40 mg/kg body weight, approximately 1.4 mg). Tumors were then excised, weighed, and preserved in buffered formalin for histopathological examination. Tumor volume was determined using Vernier calipers and calculated according to Eisa et al.^35^ as follows:

4 $${\text{V}}={\text{ }}\left( {{\text{L}} \times {{\text{S}}^{\text{2}}}} \right){\text{ }} \times {\text{ }}0.{\text{5}}$$

While V represents the tumor’s volume, L refers to the tumor with a long diameter, and S refers to the tumor with short diameter.

Based on the study of Schirner et al.^36^ the growth inhibition (%) was estimated by the following equation:

5 $${\text{Growth inhibition }}\left( \% \right)\,=\,{\text{1}}00{\text{ }}-{\text{ }}\left( {\frac{{\Delta T}}{{\Delta C}}~ \times {\text{1}}00} \right)$$

While ΔC represents the mean of change in tumor volume in EAC-bearing mice (control) and ΔT represents the mean of change in tumor volume in the treatment group. We stained the tumor slices (micrometer-sized) with eosin and hematoxylin examined them under a light microscope.

### Identification of *Streptomyces* sp. strain NEAA-5

Cultural and morphological characteristics, including aerial mycelium color, substrate mycelium pigmentation, and diffusible pigment production were evaluated according to the method of Shirling and Gottlieb^37^. Spore chain morphology and surface ornamentation were examined by SEM as described by El-Naggar et al.^38^. Physiological characteristics were assessed as described by El-Naggar and Abdelwahed^39^. Antimicrobial activity was determined against various Gram-positive and Gram-negative bacteria, as well as *Candida albicans* following the method of El-Naggar and Hamouda^40^.

Genomic DNA was extracted following the method of Sambrook^41^, and 16 S rDNA was amplified by PCR. The PCR product was analyzed by agarose gel electrophoresis and subsequently purified as described by El-Naggar et al.^42^. The obtained 16 S rDNA sequence was compared with reference sequences using the BLAST algorithm^43^. Phylogenetic analysis was conducted with MEGA version 11 software following the procedure of Tamura et al.^44^.

### Statistical analysis

The experimental design and experimental data analyses were performed using Design Expert 12 for Windows (https://www.statease.com/software/design-expert/). Three-dimensional surface plots were generated with STATISTICA 8 (https://www.statsoft.de/de/software/statistica). Statistical significance was assessed by ANOVA. Furthermore, statistical evaluation of the ANN model was conducted using JMP Pro 14.

**Fig. 1:**
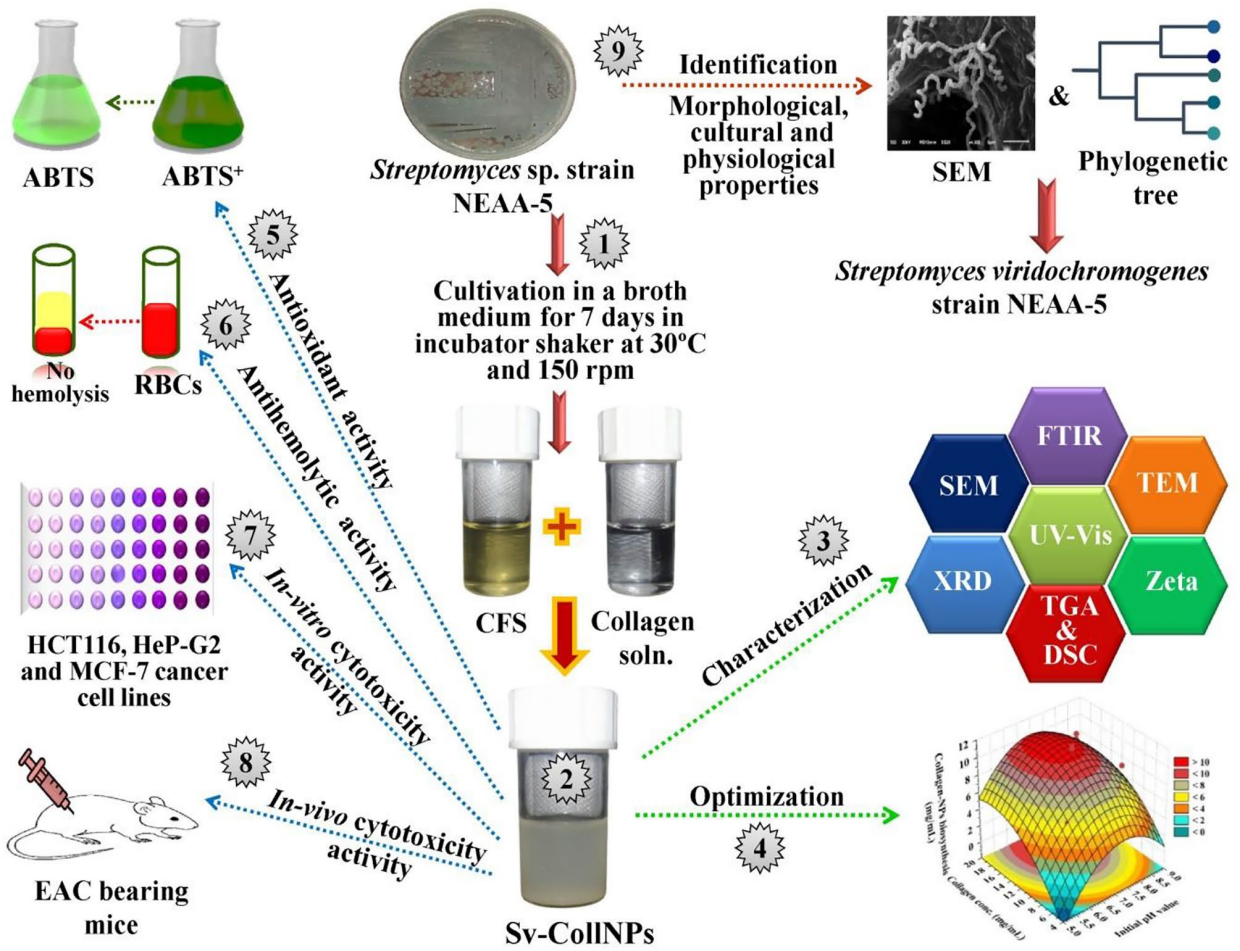
Diagrammatic outline of the systematic work processes carried out in this study.

Figure 1 presents the systematic workflow employed in this study.

## Results and discussion

### GC-MS analysis of CFS

**Fig. 2:**
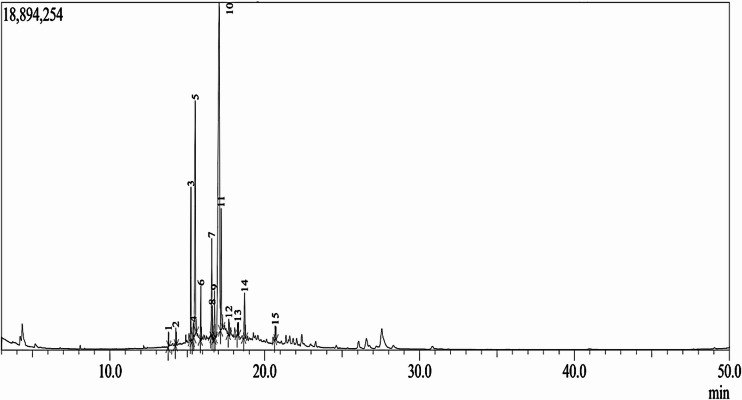
GC-MS chromatogram of the CFS of *Streptomyces* strain NEAA-5 showing fifteen major peaks.

The GC-MS profile of the CFS revealed a rich spectrum of lipid-derived metabolites, predominantly long-chain FAMEs, fatty alcohols and chlorinated lipid derivatives (Table 1; Fig. 2). These findings reflect the metabolic versatility and secondary-metabolite biosynthetic potential of *Streptomyces* species, which are well known for producing bioactive lipids and esters that contribute to antimicrobial and antioxidant activities^45^. Among the major components, saturated fatty acids such as n-hexadecanoic acid (palmitic acid) and its methyl ester, as well as octadecanoic acid (stearic acid) methyl ester, accounted for very high peak areas and heights. According to research by Fratianni et al.^46^ oleic acid, stearic acid, and palmitic acid (second degree in abundance) showed antioxidant, *in vitro* inflammatory and antibacterial action. In addition to saturated lipids, the profile included several unsaturated fatty acid methyl esters, such as cis-vaccenic acid, 9-octadecenoic acid (methyl ester) and cis-11-eicosenoic acid methyl ester. Unsaturated FAMEs are increasingly recognized for their roles in cell signaling, antioxidant activity, and interaction with biological membranes^47^. The detection of more unusual lipids, such as cyclopropaneoctanoic acid, 2-hexyl-, methyl ester, oleyl alcohol and oleoyl chloride, further reveals the biochemical diversity of CFS. Cyclopropane fatty acids have been implicated in bacterial stress adaptation and membrane fluidity regulation^48^, while fatty alcohols, and chlorinated lipids may act as biosurfactants or intermediates in antimicrobial compound biosynthesis.

**Table 1:** GC–MS quantitative analysis of the CFS of *Streptomyces* strain NEAA-5.

Peak no.	Retention time	Formula	Molecular wt. (g/mol)	Area	Name
1	13.788	C_15_H_30_O_2_	242	237,648	Methyl tetradecanoate (Myristic acid)
2	14.258	C_15_H_30_O_2_	242	210,070	Tridecanoic acid, 12-methyl-, methyl ester
3	15.232	C_17_H_34_O_2_	270	2,093,810	Hexadecanoic acid, methyl ester (Palmitic acid)
4	15.390	C_18_H_34_O_2_	282	394,153	cis-Vaccenic acid
5	15.514	C_17_H_34_O_2_	256	3,112,522	n-Hexadecanoic acid (Palmitic acid)
6	15.876	C_18_H_34_O_2_	282	335,057	Cyclopropaneoctanoic acid, 2-hexyl-, methyl ester
7	16.579	C_19_H_36_O_2_	296	936,342	9-Octadecenoic acid, methyl ester, (E)- (Elaidic acid)
8	16.617	C_19_H_36_O_2_	296	254,762	11-Octadecenoic acid, methyl ester
9	16.758	C_19_H_38_O_2_	298	855,900	Octadecanoic acid, methyl ester (Stearic acid)
10	17.060	C_18_H_34_O_2_	268	10,072,359	cis-10- Heptadecenoic acid
11	17.192	C_18_H_36_O_2_	298	1,768,122	Octadecanoic acid (Stearic acid)
12	17.684	C_20_H_38_O_2_	310	147,941	10-Nonadecenic acid, methyl ester
13	18.261	C_18_H_36_O	268	203,938	Oleyl Alcohol
14	18.701	C_21_H_40_O_2_	324	548,872	cis-11-Eicosenoic acid, methyl ester
15	20.683	C_18_H_33_ClO	300	267,993	Oleoyl chloride (Oleic acid chloride)

### Sv-CollNPs biofabrication

**Fig. 3:**
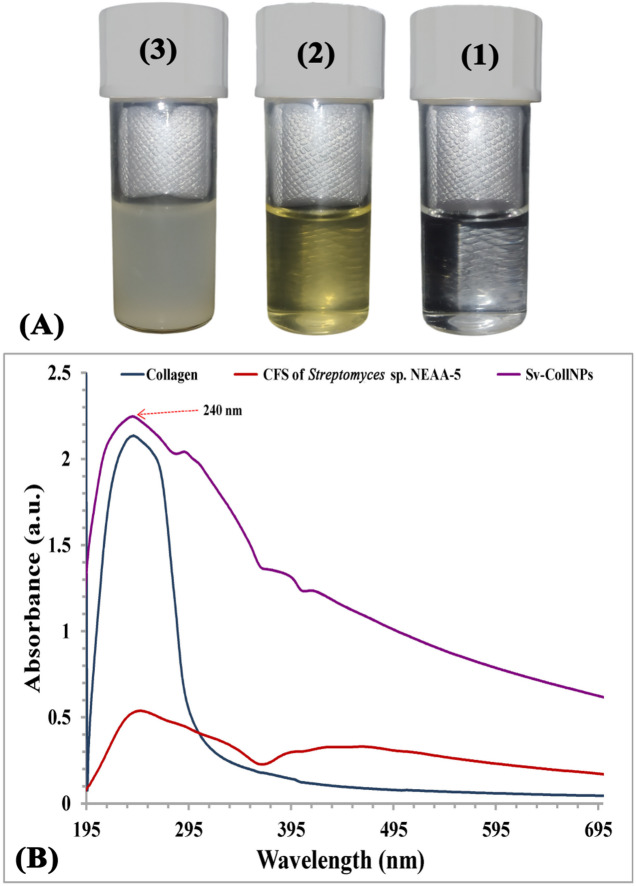
(A) Collagen solution (1), CFS of *Streptomyces* sp. NEAA-5 (2), and Sv-CollNPs (3); (B) UV– visible absorbance of Sv-CollNPs.

Sv-CollNPs were biosynthesized by mixing freshly prepared CFS with a purified marine collagen solution. As shown in Fig. 3A, the resulting Sv-CollNPs were formed through a cross-linking mechanism, which facilitated the transformation of collagen proteins into hollow spherical nanoparticles. The *Streptomyces* sp. NEAA-5 CFS is abundant in extracellular natural metabolites that function as cross-linking agents during the Sv-CollNPs biosynthesis process. These bioactive compounds including n-hexadecanoic acid (palmitic acid), hexadecanoic acid, methyl ester, octadecanoic acid (stearic acid), octadecanoic acid, methyl ester, cis-vaccenic acid, 9-octadecenoic acid, methyl ester (elaidic acid), cis-11-eicosenoic acid, methyl ester, cyclopropaneoctanoic acid, 2-hexyl-, methyl ester, oleyl alcohol, and oleoyl chloride. EDX analysis confirmed the presence of elements such as sodium, potassium, phosphorus, magnesium, chlorine, sulfur, copper, and calcium in the Sv-CollNPs sample, suggesting that their ions may act as cross-linking agents, thereby promoting Sv-CollNPs formation through collagen self-assembly.

The GC-MS spectra of *Streptomyces xinghaiensis*^12^ and *Streptomyces plicatus*^34^ contain a variety of fatty acids, such as 11-octadecenoic acid, caprylic acid, valeric acid, stearic acid, palmitic acid, palmitoleic acid, 9-octadecanoic acid, oleic acid, and elaidic acid. Besides, the fatty alcohols (as heptacosanol and cyclohexylpropyl alcohol) and fatty aldehydes (as 5-octadecenal) are also present. Natural cross-linking agents provide collagen with great flexibility and proteolytic resistance by forming Sv-CollNPs^49^. These fatty acids, alcohols, and aldehydes may act as chemical cross-linking agents for Sv-CollNPs creation and also capping and stabilizing agents. In the desolvation-based synthesis of protein nanoparticles, there are factors such as protein concentration, temperature, the type and presence of a desolvating agent (e.g., alcohol or natural salt), and the pH of the medium that play a key role in determining particle size^13^. Chemical crosslinking enhances collagen nanoparticles formation by creating covalent bonds between collagen molecules, increasing their stability, structural integrity, and resistance to degradation. This process involves introducing agents like chromium tanning, formaldehyde, carbodiimide, glutaraldehyde, and ultraviolet radiation to form stable nanostructure^50,51^.

In research conducted by Nagarajan et al.^52^, CollNPs were made from collagen solution using acetic acid, and it was also noted that the existence of calcium ions is necessary to form ionic cross-linking. Hollow nanospheres particles are formed via the self-assembly process when iron, cobalt, zinc, or copper are added to collagen peptides according to Przybyla et al.^53^. Oleic acid is the most desirable fatty acid that was employed in the production of metal NPs and is also a powerful capping agent^54^. Palmitic acid was extensively accessible and utilized as a super-hydrophobic capping agent for zinc oxide NPs^55^.

### Spectroscopy analysis of Sv-CollNPs

UV-visible studies were used to detect the maximum absorption peaks for Sv-CollNPs, collagen, and the CFS of *Streptomyces* sp. NEAA-5, as shown in Fig. 3B. At 240 nm, the marine collagen solution’s maximum absorbance peak was recorded. The maximum peak of absorption for Sv-CollNPs was detected at 240 nm, indicating the presence of collagen. Two highest absorption peaks were detected at 240 and 220 nm of collagen (type I) with a higher purity that was isolated from *Holothuria scabra* as reported by Saallah et al.^56^. Collagen is mostly composed of glycine as well as proline/hydroxyproline, which explains why its largest absorbance peak occurs from 210 to 240 nm, verifying the purity of collagen^57^. The purity of collagen is further supported by the absence of an absorption peak near 280 nm, which is typically associated with aromatic amino acids such as phenylalanine and tyrosine^58^.

### CCRD-based optimization of Sv-CollNPs biofabrication

The optimization of process variables is a crucial step in biological processes because they have a significant impact on the downstream product’s concentration, cost, and yield^59^. The optimization of the bioprocess variables has traditionally been carried out using the one-factor-at-time method. The traditional approach of bioprocess optimization involves altering a single variable at a time, while maintaining the other variables unchanged. These traditional methods have the drawbacks of being laborious, time-consuming, and costly; particularly when dealing with many variables, they consume a lot of chemicals due to the numerous experiments needed to identify the optimal conditions. Furthermore, it neglects the influence of the interactions between the independent variables^60^.

The aforementioned drawbacks can be eliminated through optimization using a number of statistical designs, such as response surface methodology, including CCRD. Compared to the traditional method, the optimization process through statistical experimental designs offers greater efficiency and accuracy by evaluating the combined effects and interactions of multiple bioprocess variables across different levels, reducing the number of experimental runs needed for many factors, reduce time and overall costs and avoid misinterpretations.

**Table 2:** CCRD matrix illustrating the biofabrication of Sv-CollNPs as influenced by initial pH (X_1_), collagen concentration (X_2_), incubation time (X_3_), and temperature (X_4_), with both coded and actual factor levels, along with the corresponding artificial neural network (ANN) analysis results.

Std	Run	X_1_	X_2_	X_3_	X_4_	Sv-CollNPs biofabrication (mg/mL)					
						Actual	CCRD		ANN		
							Predicted	Residuals	Predicted	Residuals	Validation
17	1	-2	0	0	0	4.91	5.09	-0.18	4.91	0.00	Training
5	2	-1	-1	1	-1	6.11	5.88	0.23	6.11	0.00	Training
8	3	1	1	1	-1	9.80	9.71	0.09	9.80	0.00	Training
1	4	-1	-1	-1	-1	5.66	5.84	-0.18	5.66	0.00	Training
20	5	0	2	0	0	10.92	10.92	0.00	10.68	-0.24	Validation
3	6	-1	1	-1	-1	10.50	10.30	0.20	10.50	0.00	Training
2	7	1	-1	-1	-1	7.10	7.00	0.10	7.10	0.00	Training
14	8	1	-1	1	1	5.92	5.94	-0.02	5.92	0.00	Training
16	9	1	1	1	1	7.82	7.77	0.05	7.82	0.00	Training
13	10	-1	-1	1	1	4.60	4.52	0.08	4.60	0.00	Training
23	11	0	0	0	-2	9.82	9.76	0.06	9.82	0.00	Training
7	12	-1	1	1	-1	8.92	9.05	-0.13	8.92	0.00	Training
21	13	0	0	-2	0	8.99	8.96	0.03	8.99	0.00	Training
24	14	0	0	0	2	7.11	7.22	-0.11	7.11	0.00	Training
25	15	0	0	0	0	11.82	11.66	0.16	11.66	-0.16	Validation
9	16	-1	-1	-1	1	5.33	5.24	0.09	5.33	0.00	Training
11	17	-1	1	-1	1	9.23	9.19	0.04	9.23	0.00	Training
10	18	1	-1	-1	1	6.35	6.34	0.01	6.53	0.18	Validation
15	19	-1	1	1	1	7.25	7.17	0.08	7.25	0.00	Training
29	20	0	0	0	0	11.70	11.66	0.04	11.66	-0.04	Training
30	21	0	0	0	0	11.51	11.66	-0.15	11.66	0.15	Validation
12	22	1	1	-1	1	9.42	9.47	-0.05	9.48	0.06	Validation
19	23	0	-2	0	0	4.58	4.63	-0.05	4.58	0.00	Training
18	24	2	0	0	0	6.98	6.85	0.13	6.95	-0.03	Validation
26	25	0	0	0	0	11.76	11.66	0.10	11.66	-0.10	Training
27	26	0	0	0	0	11.56	11.66	-0.10	11.66	0.10	Training
4	27	1	1	-1	-1	10.43	10.64	-0.21	10.43	0.00	Training
28	28	0	0	0	0	11.62	11.66	-0.04	11.66	0.04	Training
6	29	1	-1	1	-1	7.21	7.37	-0.16	7.21	0.00	Training
22	30	0	0	2	0	7.23	7.31	-0.08	7.23	0.00	Training

Variable	Code	Coded and actual levels				
		-2	-1	0	1	2
Initial pH level	X_1_	5	6	7	8	9
Collagen conc. (mg/mL)	X_2_	4	8	12	16	20
Incubation time (h)	X_3_	24	48	72	96	120
Temperature (°C)	X_4_	25	30	35	40	45

The CCRD technique was applied for detecting the optimum values of the four factors: initial pH level (X_1_), collagen concentrations (X_2_), incubation time (X_3_), and temperature (X_4_), as well as their interactions’ influences on Sv-CollNPs. Table 2 displays the CCRD design matrix in addition to the results of 30 experimental trials. The central experimental trial was repeated six times (15, 20, 21, 25, 26, and 28). Table 2 also exhibits the predicted and observed values of Sv-CollNPs biofabrication for various combinations of the four independent factors.

The findings of this study demonstrated that the biofabrication of Sv-CollNPs was strongly impacted by the values of the four variables. The highest Sv-CollNPs biofabrication (11.82 mg/mL) was produced in run number 15, which is one of the central runs. This was attained with an initial pH level (7), collagen concentrations (12 mg/mL), incubation time (72 h), and temperature (35 °C). Additionally, the lowest Sv-CollNPs biofabrication (4.58 mg/mL) was produced in run number 23. This was attained when the initial pH level was 7, the collagen concentration was 4 mg/mL, the incubation time was 72 h, and the temperature was 35 °C.

### Statistical evaluation using multiple regression analysis and analysis of variance (ANOVA)

ANOVA and regression analysis were performed to evaluate the relationship between Sv-CollNPs biofabrication and the selected independent factors. Table 3 presents the ANOVA results for Sv-CollNPs biofabrication, considering the quadratic, interaction, and linear effects of four process factors: pH level (X_1_), collagen concentration (X_2_), incubation time (X_3_), and temperature (X_4_). Model validation was assessed using the data in Table 3, which included coefficient estimates, *P*-values (probability), *F*-values (Fisher), predicted R^2^, adjusted R^2^, and R^2^. The model’s reliability was further verified by evaluating the lack-of-fit test.

R^2^ is a statistical measure that indicates the proportion of variance in the response variable explained by the independent factors and their interactions. The chosen model displayed an R^2^-value of 0.9976 (Table 3), revealed that the model accurately depicted the real relationship among the variables under investigation and the response values. An R^2^ value of 0.9976 illustrates that 99.76% of the variation in Sv-CollNPs biofabrication could be due to the independent variables and explained by the model. Only 0.24% of the total variation in Sv-CollNPs biofabrication remained unexplained by the model. Considering this, the current R^2^-value suggested that the model is reliable for Sv-CollNPs biofabrication in the current investigation. The model also showed excellent agreement between the observed and predicted Sv-CollNPs biofabrication. The coefficient of determination (R^2^) ranges from 0 to 1, where 0 indicates no correlation and 1 represents a perfect fit. Regression models with R^2^ values above 0.9 are generally regarded as having a very high degree of correlation^61^. In addition, the adjusted R^2^ = 0.9954 was very high, further confirming the strong significance of the model for studying Sv-CollNPs biofabrication (Table 3). The values of predicted-R^2^ and adjusted-R^2^ should fall within a 20% range of one another, indicating that the model demonstrates both high significance and accuracy, as well as a satisfactory level of concordance between the two metrics^62^. In this study, the predicted R^2^ (0.9882) closely matched the adjusted R^2^ (0.9954), confirming strong agreement between the predicted and experimental values for Sv-CollNPs biofabrication. Predicted R^2^ assesses the model’s predictive capability, reflecting its ability to estimate response values under varying experimental conditions^27^. The high predicted R^2^ value (0.9882) highlights the robustness of the model in predicting Sv-CollNPs biofabrication.

**Table 3:** ANOVA for Sv-CollNPs biofabrication under varying pH, collagen concentration, incubation time, and temperature.

Source of variance		Coefficient estimate	Sum of Squares	Degrees of Freedom	Mean square	F-value	*P*-value
Intercept		11.66	168.26	14	12.02	452.49	< 0.0001*
Linear effect	X_1_	0.44	4.67	1	4.67	175.92	< 0.0001*
	X_2_	1.57	59.44	1	59.44	2237.83	< 0.0001*
	X_3_	-0.41	4.09	1	4.09	154.06	< 0.0001*
	X_4_	-0.63	9.66	1	9.66	363.86	< 0.0001*
Interaction effect	X_1_ × _2_	-0.21	0.68	1	0.68	25.78	0.0001*
	X_1_ × _3_	0.08	0.10	1	0.10	3.92	0.0665
	X_1_ × _4_	-0.02	0.00	1	0.00	0.15	0.7067
	X_2_ × _3_	-0.32	1.68	1	1.68	63.38	< 0.0001*
	X_2_ × _4_	-0.13	0.26	1	0.26	9.89	0.0067*
	X_3_ × _4_	-0.19	0.60	1	0.60	22.47	0.0003*
Quadratic effect	X_1_^2^	-1.42	55.56	1	55.56	2091.69	< 0.0001*
	X_2_^2^	-0.97	25.91	1	25.91	975.58	< 0.0001*
	X_3_^2^	-0.88	21.34	1	21.34	803.28	< 0.0001*
	X_4_^2^	-0.79	17.26	1	17.26	649.75	< 0.0001*
Error effect	Lack of Fit		0.33	10	0.03	2.29	0.186
	Pure error		0.07	5	0.01		
R^2^	0.9976	Std. Dev.	0.16				
Adj R^2^	0.9954	Mean	8.41				
Pred R^2^	0.9882	C.V. %	1.94				
Adeq Precision	62.00	PRESS	1.99				

* *F*: Fishers’s test, *P*: significance, C.V: variation coefficient.

Interactions between two variables can occur in two ways: antagonistically or synergistically. Negative coefficient values indicate an antagonistic relationship, whereas positive coefficient values reflect a synergistic interaction. A positive coefficient for a given variable indicates that the yield increases with rising values of that variable, whereas a negative coefficient suggests that higher production occurs at lower values^63^. A large value of a coefficient, regardless of its sign, reflects a strong influence of the variable on the response, while values close to zero indicate minimal or negligible impact. Based on the coefficient values obtained, both initial pH and collagen concentration exhibited positive effects, enhancing the biofabrication of Sv-CollNPs. Whereas, the negative coefficients values of incubation time and temperature indicate that these variables had negative effects on Sv-CollNPs biofabrication (Table 3). Also, the mutual interaction effects between X_1_ and X_3_ had positive effects on Sv-CollNPs biofabrication (Table 3). The negative coefficient values for X_1_X_2_, X_1_X_4_, X_2_X _3_, X_2_X _4_, X_3_X_4_ interaction effects between variables and for the quadratic effects of the process variables (X_1_^2^, X_2_^2^, X_3_^2^ and X_4_^2^) indicate that these factors exert a negative effect on Sv-CollNPs biofabrication (Table 3).

*F*-values and *P-*values (Table 3) were calculated to evaluate the significance of each coefficient and the interactions among the studied factors. Factors with *P*-values ≤ 0.05 were considered to have a statistically significant effect on the response^64^. The model exhibited a highly significant fit, with an *F*-value of 452.49 and a *P*-value < 0.0001. The biofabrication of Sv-CollNPs using the CFS of *Streptomyces* sp. NEAA-5 is significantly influenced by the linear effects of the four variables under investigation (temperature, incubation time, collagen concentrations, and initial pH level), as demonstrated by the *P*-values < 0.0001 (Table 3). The *F*-values for X_1_, X_2_, X_3,_ and X_4_ are 175.92, 2237.83, 154.06, and 363.86; respectively. The mutual interaction’s effects between X_1_X_2_, X_2_X_3_, X_2_X_4_ and X_3_X_4_ (*P*-values < 0.5) have significant effects on the biofabrication of Sv-CollNPs. Conversely, the interaction effects of X_1_X_3_ and X_1_X_4_, with *P*-values of 0.0665 and 0.7067, respectively, are not statistically significant. This suggests that these interactions didn’t significantly enhance Sv-CollNPs biofabrication. The *P*-values indicate that the quadratic effects of all four variables (temperature, incubation time, collagen concentrations, and initial pH level) are statistically significant (*P*-value < 0.0001). This suggests that they have substantial impacts on the biofabrication of collagen nanoparticles. Adequate precision is valued at 62. Adequate precision assesses the signal-to-noise ratio, with values above 4 indicating that the model possesses acceptable accuracy and reliability^65^. The values of standard deviation, mean, percentage for C.V., and PRESS are 0.16, 8.41, 1.94, and 1.99, respectively.

The maximum predicted yield of Sv-CollNPs (Y) was obtained through the following second-order polynomial equation:

6 $$\begin{aligned} {\text{Y}} & \,=\,{\text{11}}.{\text{66}}\,+\,0.{\text{44}}{{\text{X}}_{\text{1}}}\,+\,{\text{1}}.{\text{57}}{{\text{X}}_{\text{2}}}\, - \,0.{\text{41}}{{\text{X}}_{\text{3}}}\, - \,0.{\text{63}}{{\text{X}}_{\text{4}}}\, - \,0.{\text{21}}{{\text{X}}_{\text{1}}}{{\text{X}}_{\text{2}}}\, \\ & \;\;+\,0.0{\text{8}}{{\text{X}}_{\text{1}}}{{\text{X}}_{\text{3}}}\, - \,0.0{\text{2}}{{\text{X}}_{\text{1}}}{{\text{X}}_{\text{4}}}\, - \,0.{\text{32}}{{\text{X}}_{\text{2}}}{{\text{X}}_{\text{3}}}\, - \,0.{\text{13}}{{\text{X}}_{\text{2}}}{{\text{X}}_{\text{4}}}\, - \,0.{\text{19}}{{\text{X}}_{\text{3}}}{{\text{X}}_{\text{4}}}\, \\ & \;\; - \,{\text{1}}.{\text{42}}{{\text{X}}^{\text{2}}}_{{\text{1}}}\, - \,0.{\text{97}}{{\text{X}}^{\text{2}}}_{{\text{2}}}\, - \,0.{\text{88}}{{\text{X}}^{\text{2}}}_{{\text{3}}}\, - \,0.{\text{79}}{{\text{X}}^{\text{2}}}_{{\text{4}}} \\ \end{aligned}$$

Where X_1_–X_4_ represents the process variables.

The fit summary results were used to identify the most suitable polynomial model for Sv-CollNPs production. Model selection was based on the highest adjusted and predicted R^2^ values. The lack-of-fit test determined models with an insignificant lack of fit. The quadratic model was highly significant (*P* < 0.0001) and effectively described Sv-CollNPs biofabrication. Its lack of fit was insignificant (*F*-value = 2.29, *P*-value = 0.186) (Table 4), confirming model validity. Compared to linear and 2FI models, the quadratic model demonstrated superior performance, with higher R² (0.9976), predicted R^2^ (0.9882), and adjusted R^2^ (0.9954), as well as a minimal standard deviation (0.16) and low PRESS value (1.99), indicating high accuracy and reliability^66^.

**Table 4:** Fit summary for CCRD results for Sv-CollNPs biofabrication.

Source	Sum of squares	Df	Mean square	F-value	*P*-value Prob > F
Lack of fit tests					
Linear	90.72	20	4.54	318.17	< 0.0001*
2FI	87.39	14	6.24	437.82	< 0.0001*
Quadratic	0.33	10	0.03	2.29	0.186
Sequential model sum of squares					
Linear vs. Mean	77.87	4	19.47	5.36	0.0029
2FI vs. Linear	3.34	6	0.56	0.12	0.9925
Quadratic vs. 2FI	87.06	4	21.76	819.41	< 0.0001

Model summary statistics					
Source	Standard deviation	R^2^	Adjusted R^2^	Predicted R^2^	PRESS
Linear	1.91	0.4617	0.3756	0.3448	110.5
2FI	2.15	0.4815	0.2086	0.1272	147.21
Quadratic	0.16	0.9976	0.9954	0.9882	1.99

Fit summary					
Source	Sequential *P*-value	Lack of Fit *P*-value	Adjusted R^2^	Predicted R^2^	
Linear	0.0029	< 0.0001*	0.3756	0.3448	
2FI	0.9925	< 0.0001*	0.2086	0.1272	
Quadratic	< 0.0001*	0.186	0.9954	0.9882	Suggested

* Significant values; df: degrees of freedom; PRESS: prediction error sum of squares; 2FI: two-factor interaction.

### Verifying the model’s accuracy

**Fig. 4:**
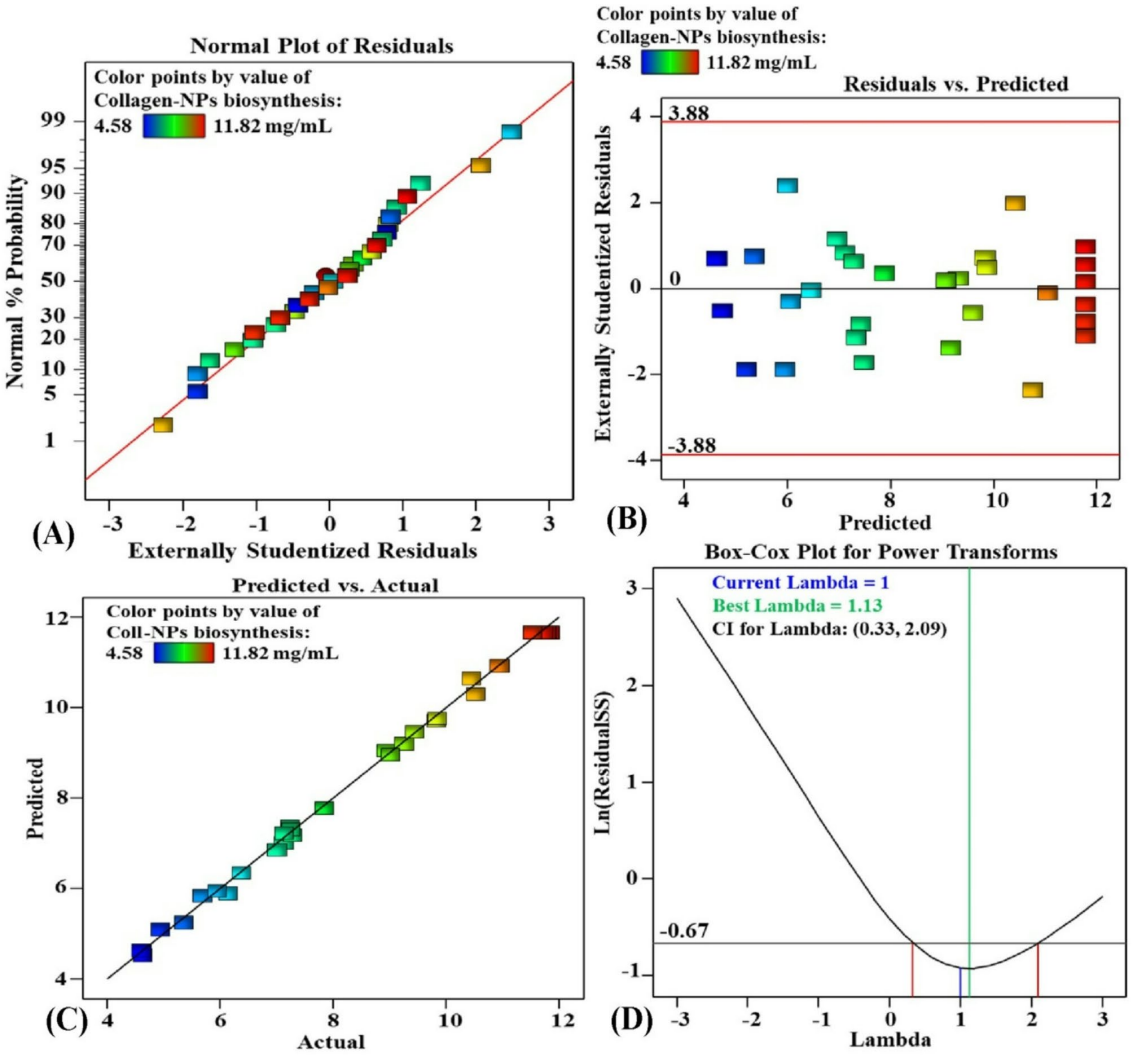
(A) NPP of residuals, (B) plot residuals versus predicted values, (C) plot of actual versus predicted values, and (D) Box-Cox plot for power transformations of Sv-CollNPs biofabrication.

Figure 4A depicts the normal probability plot (NPP) of the residuals, which was used to assess the precision and adequacy of the model. The NPP is a key diagnostic tool for evaluating whether residuals follow a normal distribution^67^. When residuals align closely with the reference line, they are considered normally distributed, while deviations from this line indicate departures from normality. In this study, the NPP reveals that the residuals followed a normal distribution and are situated close to the diagnostic line of the predicted Sv-CollNPs biofabrication, confirming the model’s validity.

Figure 4B displays a graph illustrating the relationship between the residuals and predicted values of Sv-CollNPs biofabrication using the CFS of *Streptomyces* sp. NEAA-5. A uniform and random distribution is displayed by the residuals on the graph. They are evenly distributed both above and under the zero line, and there is no obvious pattern within them, which indicates that the residuals have a consistent variance and validates the validity of the model^68^.

Figure 4C presents a comparison between the predicted and actual results of Sv-CollNPs biofabrication. The distribution of data points around the line of best fit demonstrates a strong agreement between the predicted and experimental values. The residuals have constant variance around the zero line, with no discernible pattern, confirming the model’s validity^68^.

Figure 4D shows the Box-Cox transformation plot, which is a useful tool for analyzing data that may deviate from normality. In the plot, the blue line represents the current transformation (λ = 1), while the green line indicates the optimal lambda value (λ = 1.13). The red lines mark the lower and upper bounds of the 95% confidence interval (0.33 and 2.09, respectively). As shown in Fig. 4D, the optimal lambda (λ) value falls within the range of the two vertical red lines, indicating that the model is in the optimal zone. This suggests that data transformation is unnecessary and that the model aligns well with the actual experimental results^34^.

### Three-dimensional surface plots

**Fig. 5:**
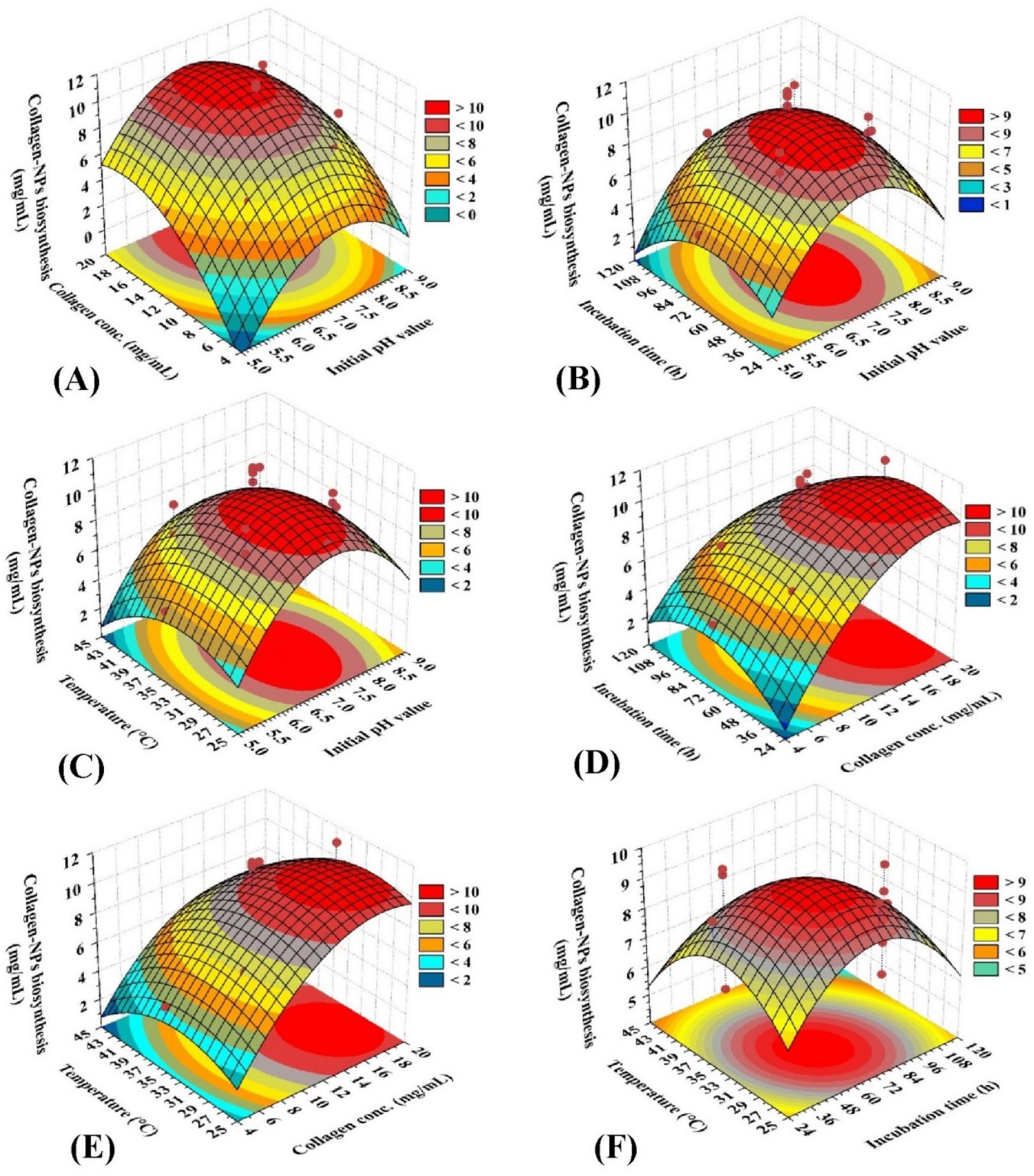
A 3D plots showing the mutual effects of initial pH level (X_1_), collagen concentrations (X_2_), incubation time (X_3_), and temperature (X_4_) on Sv-CollNPs biofabrication.

The 3D (three-dimensional) surface plots have been constructed for demonstrating the correlation between the response (Y: Sv-CollNPs biofabrication and the four independent variables being studied (X_1_: initial pH level, X_2_: collagen concentrations, X_3_: incubation time, and X_4_: temperature) as well as their interactions. These plots were used to determine the optimal levels of the independent variables for maximizing Sv-CollNPs biofabrication and to examine variations in the response. The 3D surface plots were generated by plotting Sv-CollNPs biofabrication on the Z-axis against two independent variables at a time, while keeping the remaining two variables fixed at their central levels. This approach resulted in six 3D plots representing all pairwise combinations of the four independent variables (Fig. 5A–F).

Figure 5A demonstrates the effects of initial pH level (X_1_), collagen concentrations (X_2_) on Sv-CollNPs biofabrication while maintaining incubation time (X_3_), and temperature (X_4_) at their center point levels. Figure 5A demonstrates that Sv-CollNPs biofabrication was reduced at both high and low levels of initial pH level. The maximum Sv-CollNPs biofabrication was attained when the initial pH level reached their center point levels.

Figure 5A illustrates that the biofabrication of Sv-CollNPs increases with increasing the concentration of collagen until it reaches its peak and then declines at higher collagen concentrations. To estimate the optimum values of the tested factors for the highest predicted Sv-CollNPs biofabrication, the Design Expert program (version 12) point prediction tool was utilized. Applying an initial pH level of 7.06 and a collagen concentration of 14.76 mg/mL, while keeping the incubation period and temperature at their respective center point values (at 72 h and 35 °C, respectively), will result in the maximum Sv-CollNPs biofabrication of 12.30 mg/mL.

Figure 5B shows how the initial pH level (X_1_), incubation time (X_3_) affect Sv-CollNPs biofabrication while keeping collagen concentration (X_2_), and temperature (X_4_) constant at their center point levels. Figure 5B shows that Sv-CollNPs biofabrication declined at both high and low initial pH values and incubation time. Sv-CollNPs biofabrication achieved its peak when the initial pH level and incubation time reached their respective midpoints. Using the Design Expert software point prediction tool, the ideal values of the variables were found for the maximum predicted Sv-CollNPs biofabrication. If the collagen concentrations and temperature are maintained at their respective center point levels (at 12 mg/mL and 35 °C, respectively), the maximum Sv-CollNPs biofabrication of 11.74 mg/mL can be obtained by applying an initial pH level of 7.15 and an incubation time of 66.54 h.

Figure 5C demonstrates the effects of initial pH level (X_1_), temperature (X_4_) on Sv-CollNPs biofabrication while maintaining collagen concentrations (X_2_) and incubation time (X_3_) at their center point levels. Figure 5C demonstrates that Sv-CollNPs biofabrication was reduced at both high and low levels of initial pH level and temperature. The maximum Sv-CollNPs biofabrication was attained when the initial pH level and temperature reached their center point levels. Thus, the point prediction tool of Design Expert software was used to estimate the optimum values of the variables for the maximum predicted Sv-CollNPs biofabrication. The maximum Sv-CollNPs biofabrication of 11.82 mg/mL can be achieved by applying an initial pH level of 7.17 and a temperature of 33 °C when maintaining the collagen concentrations and temperature at their respective center point levels (at 12 mg/mL, 72 h).

Figure 5D demonstrates the effects of collagen concentrations (X_2_) and incubation time (X_3_) on Sv-CollNPs biofabrication while maintaining initial pH level (X_1_) and temperature (X_4_) at their center point levels. Figure 5D reveals that the biofabrication of Sv-CollNPs rises as the concentration of collagen increases until it reaches its maximum point and subsequently decreases at higher concentrations of collagen. Additionally, Fig. 5D shows that the biofabrication of Sv-CollNPs was decreased at both high and low incubation times. When the incubation time reached its center point level, the greatest amount of Sv-CollNPs biofabrication was achieved. Thus, the point prediction tool of Design Expert software was used to estimate the optimum values of the variables for the maximum predicted Sv-CollNPs biofabrication. The maximum Sv-CollNPs biofabrication of 12.43 mg/mL was reached by maintaining the initial pH level and temperature at their center point levels (at 7 and 35 °C, respectively) and employing a collagen concentration of 15.37 mg/mL for an incubation time of 62.2 h.

Figure 5E demonstrates the effects of collagen concentrations (X_2_) and temperature (X_4_) on Sv-CollNPs biofabrication while maintaining initial pH level (X_1_) and incubation time (X_3_) at their center point levels. Figure 5E reveals a positive correlation between collagen concentrations and Sv-CollNPs biofabrication, as the increase in the collagen concentrations resulted in increasing Sv-CollNPs biofabrication until it reached its maximum. However, once the collagen concentration reached an optimum level, further increases in the collagen concentrations decreased the Sv-CollNPs biofabrication. Furthermore, Fig. 5E reveals that the biofabrication of Sv-CollNPs was reduced at both high and low temperatures. When the temperature reached its centre point, the highest Sv-CollNPs NPs biofabrication was achieved. Thus, the Design Expert software point prediction tool was employed to determine the optimal factor values for Sv-CollNPs biofabrication. The highest predicted Sv-CollNPs biofabrication of 12.47 mg/mL could be achieved by using the optimal levels of both a collagen concentration of 15.29 mg/mL and a temperature of 32.7 °C when keeping the initial pH level and incubation time at their center point levels (at 7 and 72 h; respectively).

Figure 5F demonstrates the effects of incubation time (X_3_) and temperature (X_4_) on Sv-CollNPs biofabrication while maintaining initial pH level (X_1_) and collagen concentrations (X_2_) at their center point levels. Figure 5F demonstrates that an increase in both incubation time and temperature beyond middle levels led to an increase in the Sv-CollNPs biofabrication, and then a further increase in their levels significantly decreased Sv-CollNPs biofabrication. The point prediction tool of Design Expert software determined the optimal incubation time and temperature for Sv-CollNPs biofabrication. The maximum Sv-CollNPs biofabrication of 11.82 mg/mL can be achieved by applying the incubation time of 66.85 h and temperature of 33.2 °C while maintaining the initial pH level, and the collagen concentrations at their respective center point levels (at 7 and 12 mg/mL, respectively).

### Desirability function (DF)

**Fig. 6:**
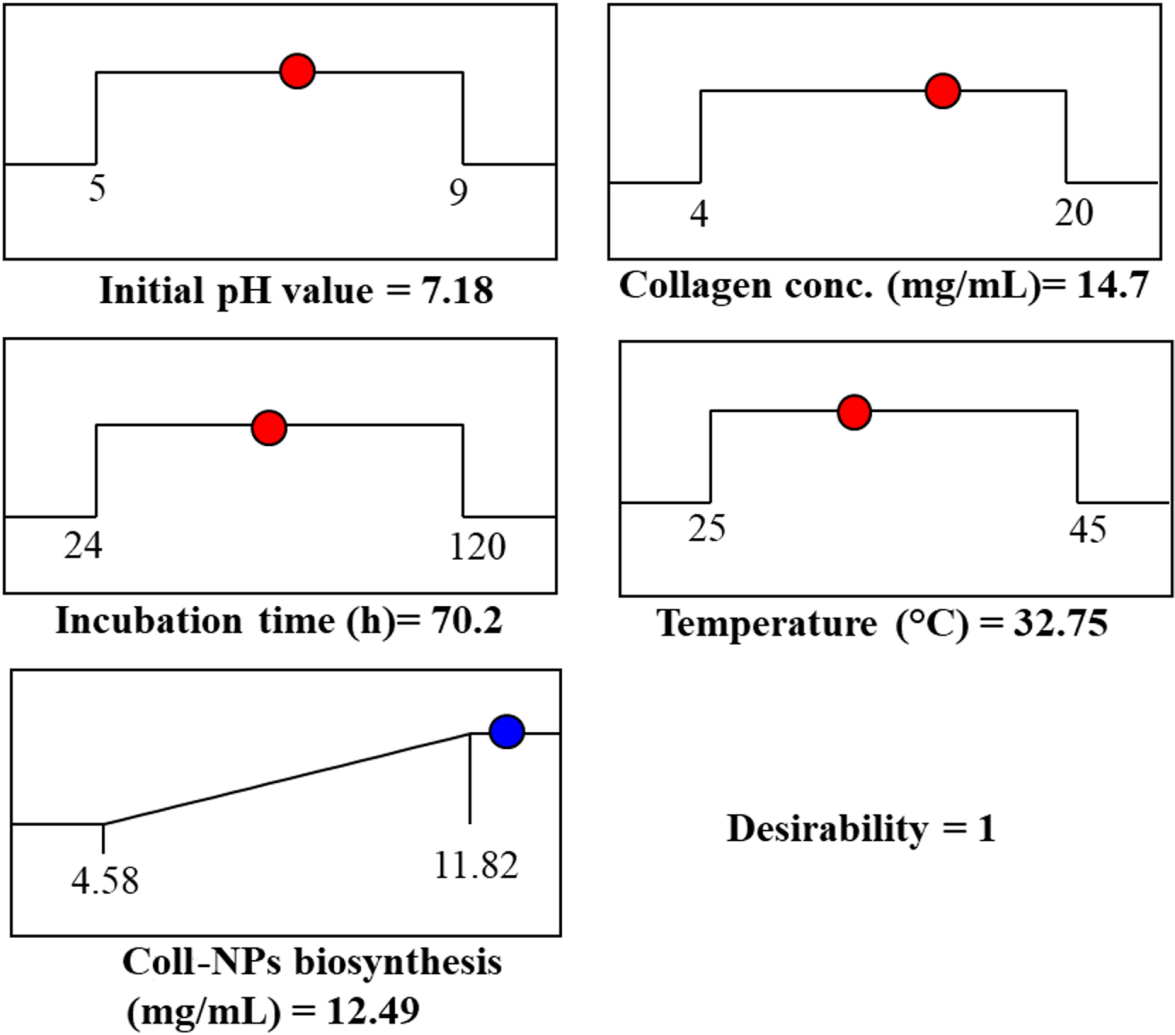
The optimal predicted conditions for maximum Sv-CollNPs biofabrication and the desirability value.

The optimal predicted conditions for maximum Sv-CollNPs biofabrication were determined using the desirability function tool, as illustrated in Fig. 6. The value of the desirability function is often predicted theoretically before the optimization process is validated experimentally^69^.

The optimal predicted conditions for maximum Sv-CollNPs biofabrication (12.45 mg/mL) were an incubation time of 70.02 h, a temperature of 32.75 °C, a collagen concentration of 14.7 mg/mL, and an initial pH of 7.18. For the model verification, an experiment was performed under these predicted conditions. The maximum experimental Sv-CollNPs biofabrication was 12.17 mg/mL, closely matching the predicted value. This verification confirms that the DF accurately predicts the optimal process variable values, demonstrating the model’s high reliability and precision.

### The performance of the predictive models

**Fig. 7:**
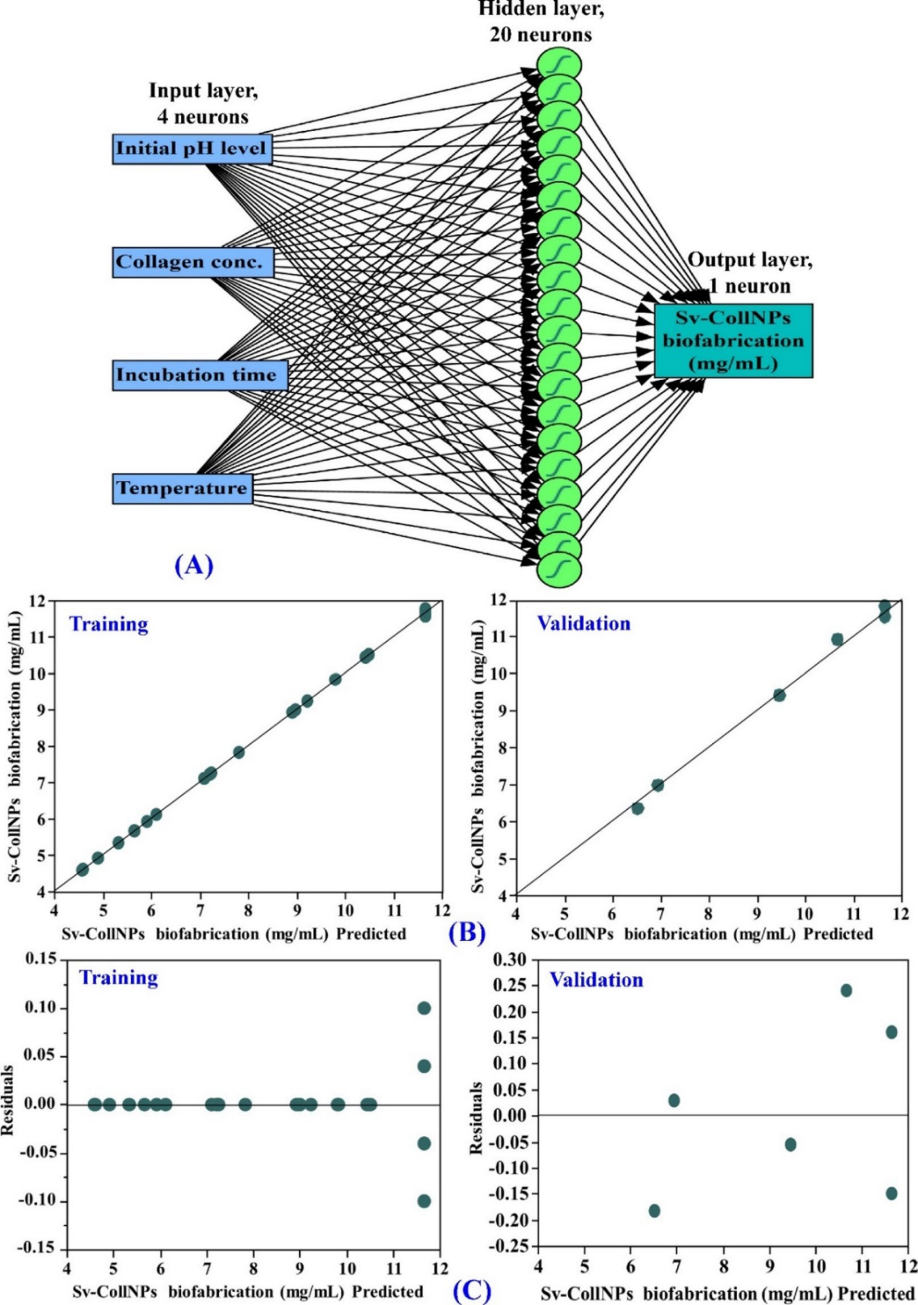
ANN for Sv-CollNPs biofabrication by *Streptomyces* sp. NEAA-5 (A), the ANN actual versus predicted Sv-CollNPs biofabrication (B), and ANN predicted Sv-CollNPs biofabrication versus the residuals (C).

To assess the effectiveness of the optimization strategy, both CCRD and ANN models were developed and evaluated based on statistical performance metrics. Table 5 presents the ANN analysis and a comparative evaluation of the predictive capabilities of the ANN and CCRD models for Sv-CollNPs biofabrication, influenced by initial pH (X_1_), collagen concentration (X_2_), incubation time (X_3_), and temperature (X_4_). Figure 7A illustrates the architecture of an ANN model developed to predict the microbial-mediated synthesis approach for Sv-CollNPs. The network consists of three main layers, including an input layer comprising 4 neurons, each representing one of the four independent variables, and a single hidden layer with 20 neurons. Each input neuron is fully connected to all neurons in the hidden layer. In addition to the output layer that contains a single neuron that represents the predicted Sv-CollNPs biofabrication. The dense interconnections between the layers of the network represent a fully connected multilayer architecture that relates input variables to the Sv-CollNPs fabrication process. The ANN demonstrated excellent predictive ability in predicting the biosynthetic yield of Sv-CollNPs, with a coefficient of determination (R^2^) of 0.9998 for the training dataset and 0.9948 for the validation dataset, indicating a strong correlation between the observed and predicted Sv-CollNPs biofabrication. Furthermore, the low RMSE values of 0.0311 (for training) and 0.1545 (for validation) reflect the model’s high accuracy and low prediction error. Furthermore, the MAD values of 0.0117 and 0.1363 for training and validation, respectively, support the robustness and reliability of the model. The low SSE values of 0.0232 (for training) and 0.1432 (for validation) also confirm the accuracy of the model. The LogLikelihood scores of – 49.2454 for the training set and − 2.6913 for the validation set further confirm the robustness of the ANN model, reflecting an excellent fit to the training data and reliable predictive capability for estimating Sv-CollNPs biofabrication yield under future experiments.

**Table 5:** ANN analysis and modeling comparison of predictive capability between ANN and CCRD.

Measure	ANN		Overall model performance		
	Training	Validation	Statistics	Measures of Fit for CCRD	Measures of Fit for ANN
R Square	0.9998	0.9948	R^2^	0.9976	0.9990
RMSE	0.0311	0.1545	RASE	0.1161	0.0745
Mean Abs Dev	0.0117	0.1363	AAE	0.0983	0.0366
-LogLikelihood	-49.2454	-2.6913	Freq	30	30
SSE	0.0232	0.1432			
Sum Freq	24	6			

Root mean squared error (RMSE), mean absolute deviation (MAD), sum of squared errors (SSE), root average squared error (RASE), and average absolute error (AAE).

### The comparative predictive capability of ANN and CCRD

When compared with the CCRD model, the ANN exhibited consistently better performance across all goodness-of-fit metrics (Table 5), including markedly lower RASE values (0.0745) and AAE values (0.0366), indicating greater predictive precision. These findings confirm the ANN’s strong predictive accuracy and its capacity to predict Sv-CollNPs biofabrication yield under future experiments. Overall, the results validate the ANN model’s robustness, precision, and reliability as an optimization tool for enhancing Sv-CollNPs biofabrication yield.

Figure 7B depicts the relationship between the experimental and ANN-predicted yields of Sv-CollNPs. In the training dataset, the predicted values show a strong alignment with the actual measurements, with data points closely following the diagonal line of perfect fit, demonstrating the model’s high accuracy. In the validation dataset, there is a similarly strong correlation between predicted and observed values, further confirming the model’s reliability. In both cases, the tight clustering of points along the diagonal indicates that the ANN can accurately predict Sv-CollNPs biofabrication yield from the input variables, providing accurate predictions for Sv-CollNPs biofabrication yield.

Figure 7C displays the residual plots of the ANN model for both the training and validation datasets, highlighting the distribution of prediction errors. The residuals, defined as the differences between the observed and predicted Sv-CollNPs yields, are plotted against the predicted values for both the training and validation datasets. These points are randomly dispersed around the zero line without any visible pattern. Most residuals lie within a narrow interval, reinforcing the model’s high predictive accuracy. The right panel shows the residuals for the validation dataset, which mirror the training results, with symmetrical dispersion around zero and no discernible trend. The relatively small residual values confirm the ANN model’s robustness for predicting Sv-CollNPs biofabrication yield in future experiments.

### Sv-CollNPs biofabrication yield predictions using the ANN 

**Fig. 8:**
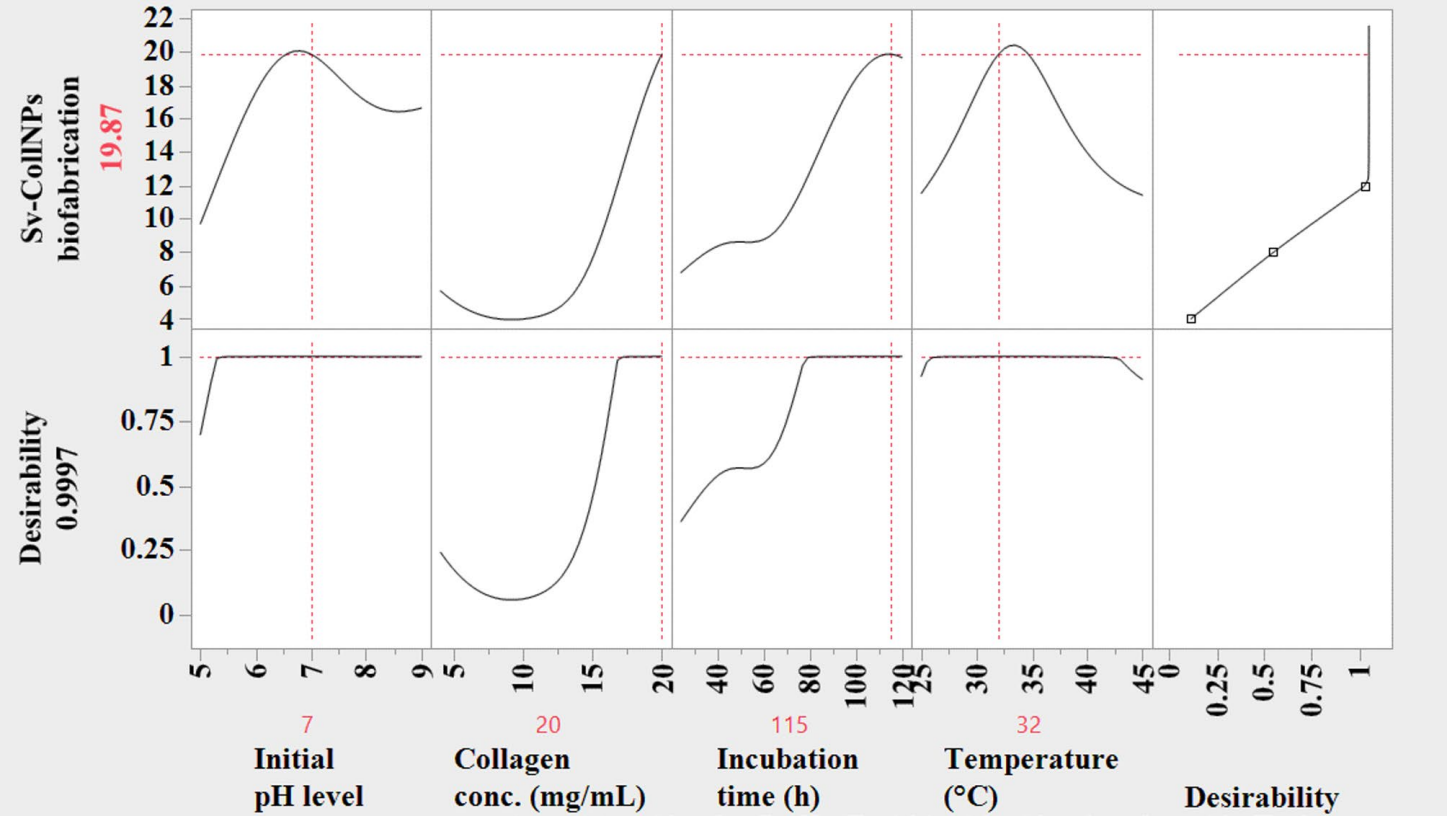
The optimization plot of DF and the ANN optimum values of predicted Sv-CollNPs biofabrication by *Streptomyces* sp. NEAA-5.

Comparative statistics indicated that the ANN provided more accurate predictions and reduced errors more effectively than the CCRD model. Consequently, the ANN was chosen as the preferred approach for optimizing Sv-CollNPs biosynthesis (Fig. 8). Optimization was conducted using the DF in JMP Pro 14, which assigns a value from 0 (least desirable) to 1 (most desirable) to estimate optimization efficiency prior to experimental confirmation. The model predicted a maximum Sv-CollNPs yield of 19.87 mg/mL with a desirability value of 0.9997, reflecting a highly reliable prediction. The optimal process parameters were determined as pH 7, collagen concentration of 20 mg/mL, incubation time of 115 h, and temperature of 32 °C. Individual desirability plots (Fig. 8) indicated that each factor had a significant influence on the Sv-CollNPs yield. The high desirability value confirmed that this parameter combination effectively maximized Sv-CollNPs production. Experimental validation supported the model’s predictive power, producing an actual Sv-CollNPs yield of 19.1 mg/mL, which closely matched the theoretical prediction.

Several previous studies have reported the successful validation and prediction of responses using ANN analysis of CCD results^31,70^. Compared to the CCD approach, ANNs demonstrated superior predictive performance and lower error values^71^.

While CCRD provided a statistically optimized conditions based on the desirability function, ANN model utilized the same experimental data to learn complex, nonlinear relationships among variables that cannot be described by a second-order polynomial regression model. ANN produced slightly different “optimal” conditions compared to those calculated by the CCRD, this difference should not be seen as a contradiction or error. The difference arises from the higher sensitivity and adaptive learning capacity of ANN which is able to build upon and improve the statistical optimization achieved by CCRD, demonstrating ANN’s superior predictive accuracy and generalization ability.

### Microscopy analysis of Sv-CollNPs

**Fig. 9:**
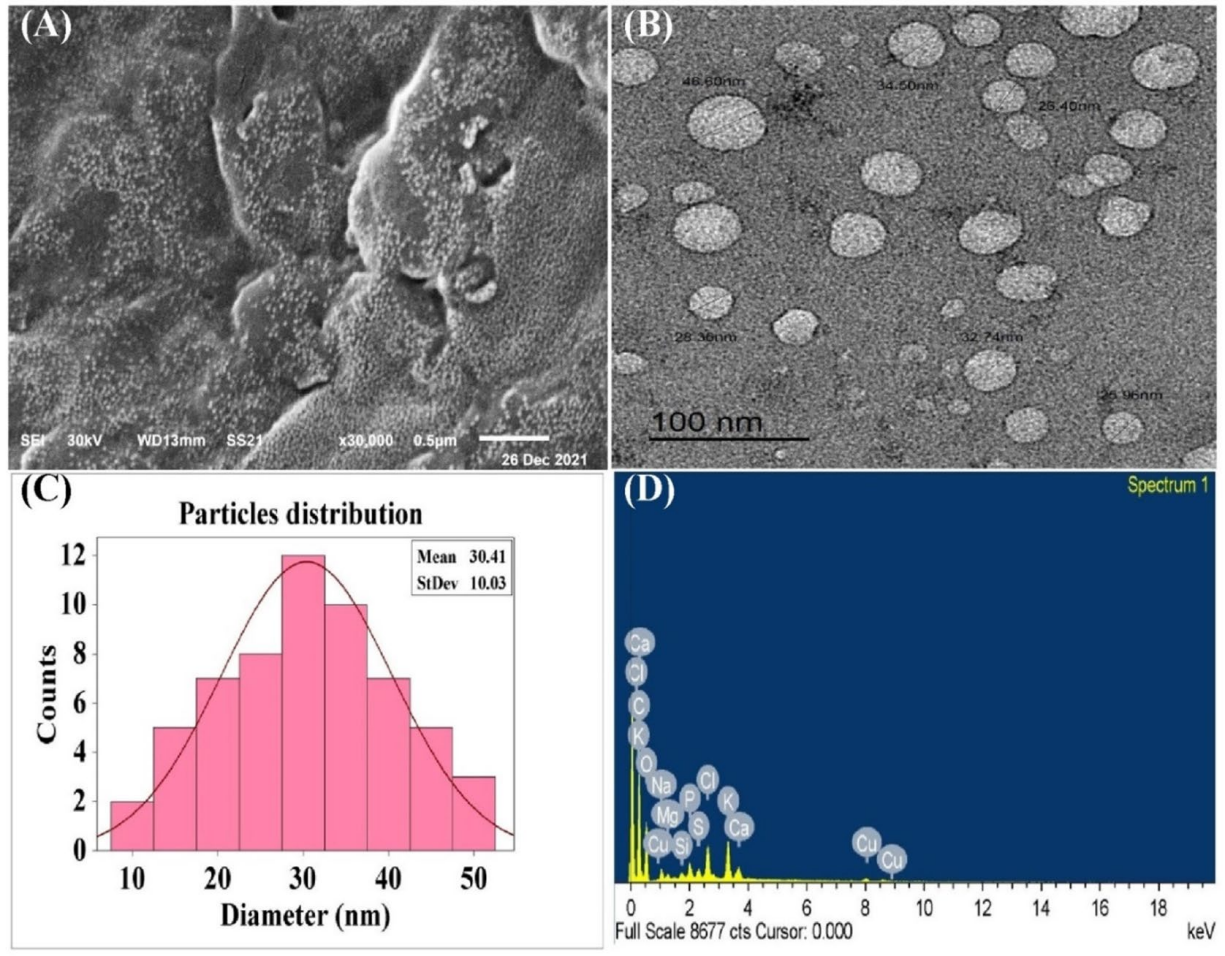
(A) SEM, (B) TEM, (C) Particle size, and (D) EDX of Sv-CollNPs.

The two most widely accepted techniques for analyzing nanoparticles are commonly recognized as SEM and TEM. Following the optimization of the biofabrication process, SEM and TEM were employed to examine the surface area, particle dimensions, and crystalline structure of the biosynthesized Sv-CollNPs. Although these two techniques differ in their imaging principles, SEM providing detailed surface morphology, whereas TEM provides high-resolution insights into internal structure. They were used in a complementary manner to obtain a comprehensive assessment of the physical characteristics of the synthesized nanoparticles^72–74^. The Sv-CollNPs’ morphology was detected and confirmed using SEM and TEM, as shown in Fig. 9A & B, respectively. Hollow sphere particles at the nanoscale were observed in the transmission electron micrograph^12,34^. The synthesized Sv-CollNPs were found to possess an average of diameter of 30.41 nm with a standard deviation of 10.03, as shown in Fig. 9C. When compared to the mean diameter of approximately 100 nm reported by Luo et al.^75^, who synthesized CollNPs through a heat-assisted chemical route, the average diameter obtained in the present study is perfect. In another report, CollNPs were synthesized using the electrospray deposition (ESD) technique, where a collagen solution in 50 v/v% acetic acid containing NaCl or CaCl_2_ produced quasi-monodispersed solid particles with average diameters of approximately 900 nm and 693 nm, respectively^52^. Similarly, collagen–poly (3-acrylamidophenylboronic acid) nanoparticles synthesized chemically exhibited a well-defined spherical morphology with a narrow particle size distribution and an average diameter of around 75 nm^76^. In contrast, biologically synthesized nanoparticles tend to exhibit smaller dimensions. For instance, collagen nanoparticles biosynthesized using the cell-free supernatant of *Streptomyces plicatus* strain NEAA-3 displayed a mean diameter of 33.15 ± 10.02 nm^34^. Additionally, hydrolyzed fish collagen nanoparticles (HFC-NPs) produced using a water-soluble extract derived from *Ulva fasciata* biomass displayed an average size of 27.25 nm^4^. These findings collectively emphasize that microbial and green synthesis approaches generally yield finer and more uniform nanoparticles compared with traditional chemical or physical methods, supporting the eco-friendly and efficient nature of biologically driven nanofabrication. The tiny size of nanoparticles is beneficial, particularly for applications in medicine. Due to their small sizes, nanoparticles have the ability to move widely throughout the human body and penetrate cells as well as attach to particular cells types. With particle sizes below 100 nm, CollNPs possess nanoscale features that could render them highly suitable as three-dimensional biomaterials^34^. Furthermore, its elevated surface area-to-volume ratio promotes effective interactions and enhances penetration into wound site^77^.

### EDX investigation

For the Sv-CollNPs powder, the EDX findings revealed elemental signals for C, O, Mg, P, Na, K, Ca, Cl, Cu, Si and S, as shown in Fig. 9D. Carbon had the highest abundance with atomic percentages of 66.29 and weights of 57.81, followed by oxygen, which has values of 36.22 and 30.18. From the copper grid, the Cu signal was received^78^. The elements that have been obtained from Sv-CollNPs biosynthesized using the CFS of *Streptomyces* sp. NEAA-5 were S, P, Cl, Mg, Na, Ca, and K. These components originated from the CFS of the *Streptomyces* sp. NEAA-5 media and appeared as traces of the media’s constituent parts (starch, MgSO_4_, KNO_3_, NaCl, K_2_HPO_4_, FeSO_4_.7H_2_O and yeast extract). Some of the trace elements that were exposed to NaCl were Ca, Cu, Mg, K, S, and P. The elements Na, Cl, Pb, NO_3_, arsenic, and SO_4_ have been combined with K_2_HPO_4_^12^.

### FTIR investigation

**Fig. 10:**
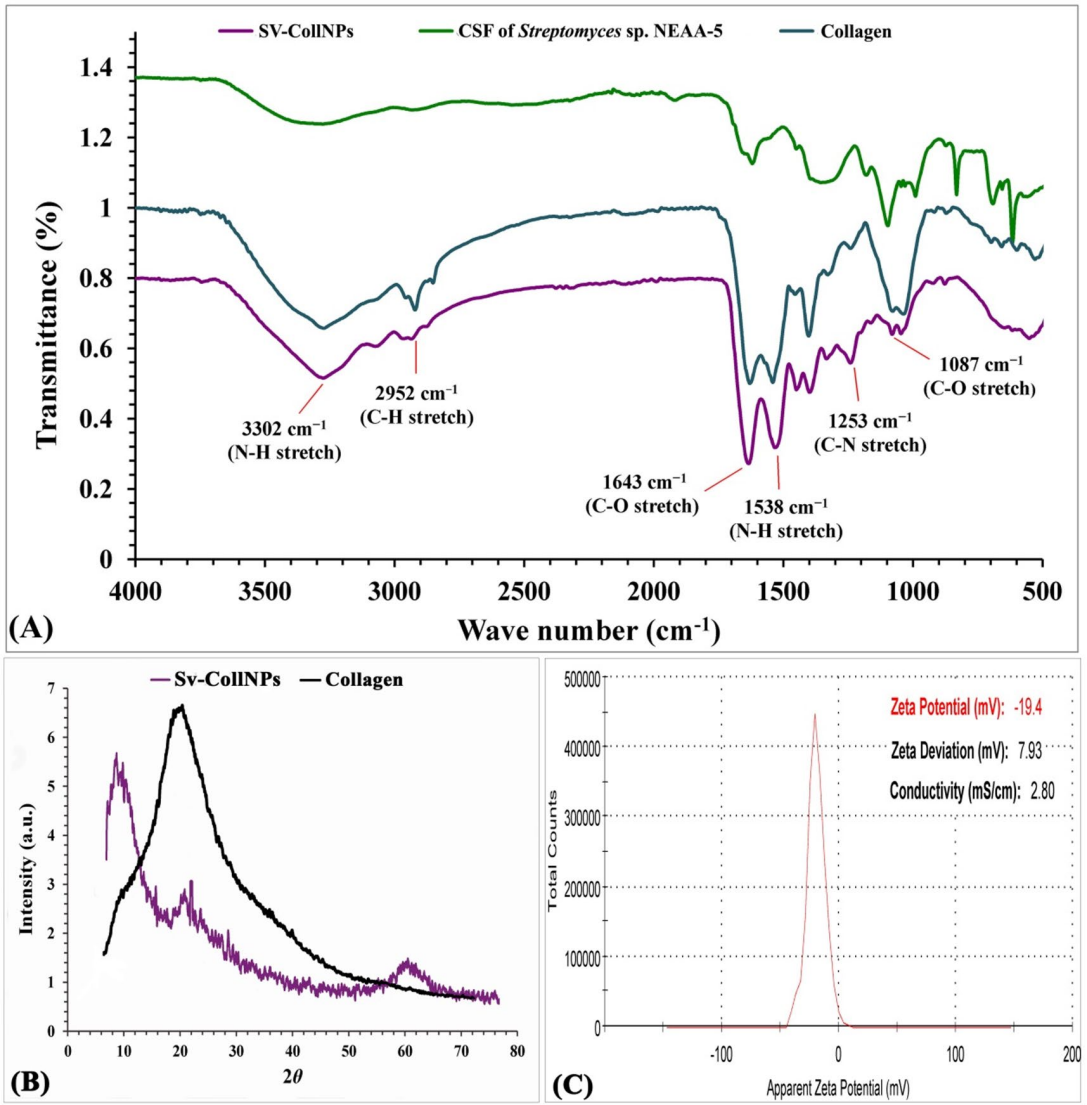
(A) FTIR spectra, (B) XRD analysis, (C) Zeta potential analysis of Sv-CollNPs.

Researchers successfully studied protein stability, conformational changes, secondary structures and structural dynamics by employing FTIR analysis^34,79^, as exhibited in Fig. 10A. The FTIR spectrum of the Sv-CollNPs generated several absorption peaks (3792, 3302, 3097, 2987, 2952, 2899, 2345, 2145, 1643, 1538, 1454, 1444, 1370, 1253, 1087, 885 and 563 cm^− 1^). The peak at 3302 cm^− 1^ represented the presence of NH stretch of NH_2_ in aromatic amines, amide A, as well as primary amines, which varied in value from 3500 cm^− 1^ to 3300 cm^− 1^^80^.

As indicated by Movasaghi et al.^81^, amide band corresponds to symmetric and asymmetric C–H stretching vibrations found in CH_3_ and CH_2_ groups of aliphatic molecules. This band is typically observed at an absorption peak near 2952 cm^− 1^, within the spectral range of 2990–2850 cm^− 1^. Haris and Severcan^82^ reported that the standard frequency marker of the peptide secondary molecule was identified among 1600 and 1700 cm^− 1^. as reported by. At 1643 cm^− 1^, the amide I bond, which is linked to the C = O along the polypeptide backbone, was observed. The peak in secondary amides at around 1538 cm^− 1^ is attributed to NH, and it falls within amide II’s standard absorption ranges from 1478 to 1565 cm^− 1^^83^. The collagen triple helical structure was verified by the finding that the absorption peak around 1253 cm^− 1^) C–N stretching( was located between the amide II group at 1411 cm^− 1^ and amide III group at 1241 cm^− 1^, which was nearly equal to 1^84^. Alcohol’s C–O stretch was demonstrated by the peak value at 1087 cm^− 1^. According to Lambert^83^, the spectral peaks observed at 885 cm^− 1^ and 563 cm^− 1^ confirmed the presence of 1,2,4-trisubstituted benzene, the Ar–OH functional group characteristic of phenols, and the O–C = O moiety associated with carboxylic acids, respectively.

### XRD investigation

Figure 10B displays the X-ray patterns of Sv-CollNPs and collagen. Sv-CollNPs exhibit a high degree of crystallinity with two distinct and strong peaks in their pattern around 2*θ* = 10° and 24°^85^. The high degree of crystallinity of the biosynthesized Sv-CollNPs give a strong peak at 2*θ* = 10°. The collagen’s triple helical structure is associated with the first peak, while the distance among skeletons is related to the second peak^34^.

### Zeta (ζ) potential analysis

The ζ-potential is required to detect the cellular interactions between molecules and charged ions, as mentioned by Chandrasekaran et al.^86^. In colloidal solutions, the nanoparticles’ stability is primarily determined by the zeta potential, which can have a positive or negative value^34^. Because of strong electrostatic repulsion among the elements, the surface charges on it may have an impact on how stable the preparation of the nanoparticle is^87^. Using ζ-potential, this charge was detected by a voltage applied throughout two electrodes on the cell’s opposite sides, which contained the dispersion of the particle. Then these charged particles are drawn to the electrode that has an opposite charge^28^.

The increase in surface area, dispersion capacity, as well as catalytic activity of marine Sv-CollNPs (type I) is due to its ζ-potential finite range^88^. As illustrated in Fig. 10C, there is only one peak in the ζ-potential pattern, which confirms that Sv-CollNPs are highly homogeneous. In addition, the measured value of ζ-potential was − 19.4 ± 0.1 mV (*n* = 3), corresponding to a Zeta deviation of 9.93 mV as well as the conductivity at 2.8 mS/cm. This ζ-potential value agrees with that of El-Sawah et al.^34^ (-19.3 mV), who used the CFS of *S. plicatus* to biosynthesize CollNPs. In liquids or even dispersions, high ζ-potentials hinder nanoparticles from gathering and additionally provide stability to molecules of small size. As a result, colloids with higher, either positive or negative ζ-potential, tend to possess greater electrostatic stability, whereas lower ζ-potential values generally promote flocculation or coagulation^27,89^.

### Thermal behavior of Sv-CollNPs

To ascertain the thermal properties regarding Sv-CollNPs generated via biofabrication, two main methods were employed: DSC alongside TGA. The thermoanalytical properties required to describe the nanoparticles’ properties that participate in chemical processes over a predetermined temperature range were measured by both TGA and DSC analyses. TGA is used to study the differential thermal analysis as a function of time in regard to mass change and/or a steady temperature. Alternatively, it can be examined in response to a sample mass loss in relation to variations in temperatures. In contrast, DSC estimates the amount of required or produced heat flow in relation to temperature variations at a specific time. A key difference between TGA and DSC is the way heat alters samples and how to detect those variations^27,90,91^.

**Fig. 11:**
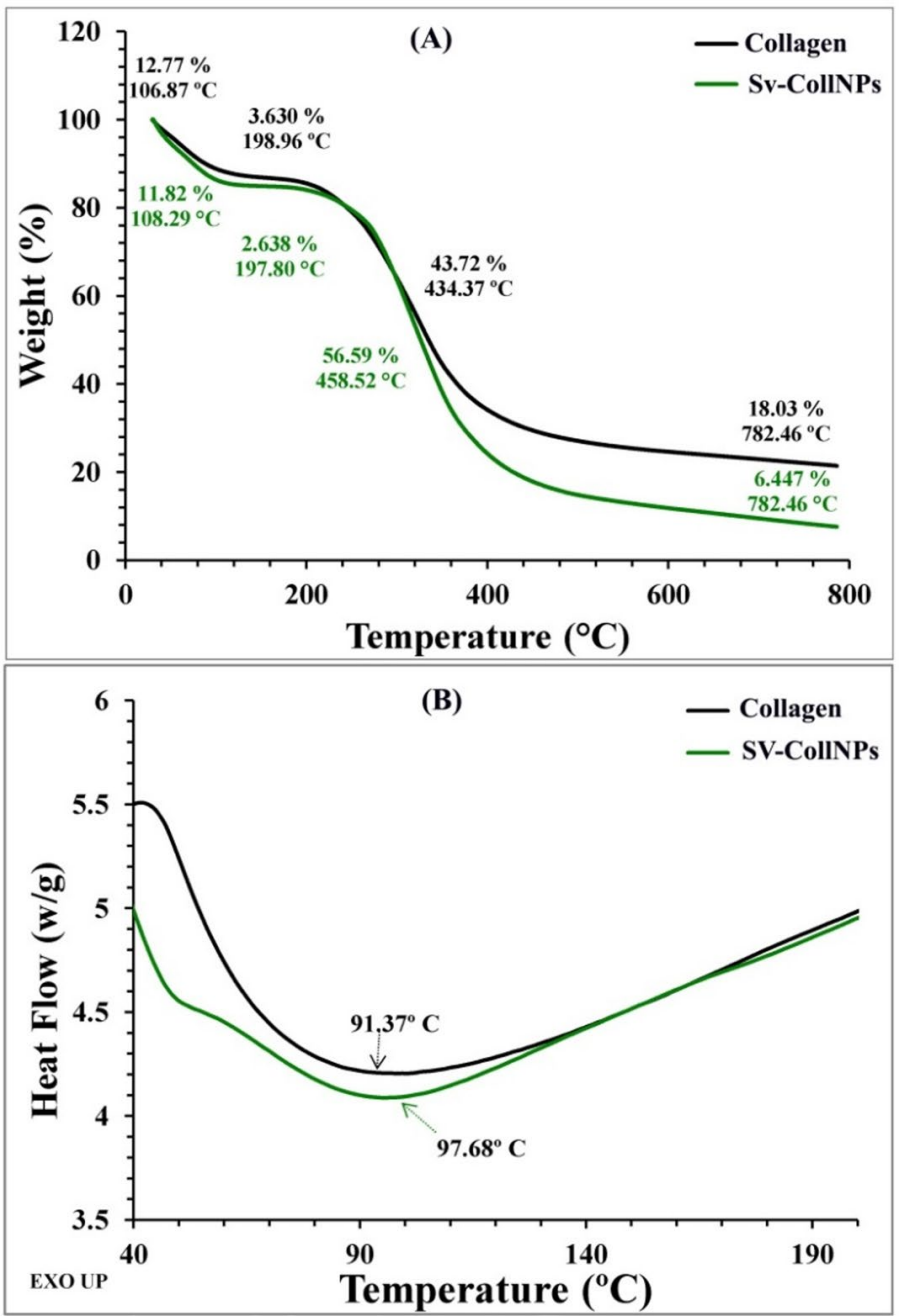
TGA (A), DSC (B) of Sv-CollNPs and collagen.

The tendency of polymeric nanoparticles to tolerate heat and preserve their characteristics, like elasticity, toughness, or strength, at a specific temperature is known as their thermal stability^92^. Sv-CollNPs and collagen are commonly tested for thermal stability using TGA and DSC, as shown in Fig. 11A and B, respectively.

The thermal properties of biosynthesized Sv-CollNPs weighting 2.362 mg were examined using a thermogravimetric analyzer, which has the TGA-50 H model. The sample was processed within ambient temperature up to 800 °C, with a rate of flow of 40 mL/min. Figure 11A presents that Sv-CollNPs underwent their first transition phase, with the particles losing weight at 108.29 °C corresponding to a weight ratio of 11.82. In comparison with collagen’s thermal degradation, the percentage of the weight became 12.77, while the transition temperature moved to 106.87 °C as presented in Fig. 11A. According to Ebnesajjad et al.^93^, the moisture amount and volatile constituents of the samples are responsible for this initial weight loss. Furthermore, El-Sawah et al.^12^ reported that the CollNPs’ polypeptide chain is believed to have broken down thermally, causing the first transition step of the material to occur. Collagen’s second temperature of transition was recorded at 198.96 °C having a percentage by weight at 3.63, whereas Sv-CollNPs’ second transformation temperature was measured at 197.80 °C having a weight% of about 2.638. Sources of the second degradation stage include the collagen breakdown as well as consuming the waste of collagen organic matrix^94^. From Fig. 11A, it is obvious that the third transition temperature is the largest for both. The collagen as well as Sv-CollNPs residual weight ratios were 43.72 and 56.59, respectively, in respect to their various transition temperatures of 434.37 and 458.52. The study’s results demonstrated that the Sv-CollNPs’ improved thermal stability was a result of their extensive surface capping agent application, which included bioactive components. Tampieri et al.^95^ state that as a result of the residual organic molecules burning, the net weight% of collagen along with Sv-CollNPs seemed about 18.03% and 6.447%, respectively, with the same conversion temperature at 782.46 °C.

A simple method that may provide details regarding the real thermodynamic characteristics of protein thermal transitions is the DSC approach^96^. In theory, details about the protein thermal transitions may be detected through estimating the heat flow through the collagen reference material and the Sv-CollNPs sample^34^. Figure 11B presents the DSC spectra of Sv-CollNPs. Sv-CollNPs along with the collagen sample’s transition temperatures were determined at the highest transition temperature (T_max_), which is further referred to as the endothermic peak of transition curves as necessary^34^. The breakdown of the triple-helical structure and the dissociation of the water molecules combined to the collagen compound are linked to the endothermic peak seen in Fig. 11B at 97.68 °C. This temperature was higher than the temperature at which collagen breaks down (91.37 °C)^97^. The increased endothermic temperature peak of CollNPs is thought to have resulted from the bioactive substances found in the capping agents, and these were isolated from the CFS of *S. plicatus*^34^.

### Anti-oxidant effect of Sv-CollNPs

Antioxidant peptides derived from natural collagen protein are widely desired for their capacity to bind with minerals, which offers fresh perspectives on avoiding and treating illnesses associated with oxidative stress^98^. The theory underlying the ABTS method states that by providing a hydrogen atom to the ABTS^+^ radicals. Antioxidants have the ability to decolorize the solution by eliminating the radical cation. The results demonstrated that Sv-CollNPs have a high degree of antioxidant effect when compared with native collagen and the standard vitamin C. At a concentration of 0.5 mg/mL, Sv-CollNPs showed 69.8 ± 1.5% radical scavenging efficiency, whereas collagen exhibited 43.1 ± 1.2%, and vitamin C (positive control) showed 88.2 ± 1.0%. All experiments were performed in triplicate (*n* = 3), and statistical analysis using one-way ANOVA revealed that the difference between Sv-CollNPs and collagen was significant (*P* < 0.05), confirming the enhanced antioxidant potential of the nanoparticle formulation. The arrangement and makeup of amino acids inside peptides are crucial factors that impact collagen’s antioxidant capacity^99^. Marine collagen contains many various amino acids and is distinguished by its elevated glycine and proline^34,100^. The higher concentration of glycine and proline in the collagen amino acid sequence, which contributes to protons’ defense against free radicals, inhibits chain reactions to free radicals^101^. An additional possibility could be that collagen peptides contain -OH and -NH_2_ groups, which have the capacity to attach to free radicals^102^.

### Anti-hemolytic effect of Sv-CollNPs

Erythrocyte oxidation is a model for biological membrane oxidation damage, and hemolysis is caused by the oxidation of chains in proteins and lipids in erythrocytes triggered by free radicals that are produced in the aqueous phase^103,104^. AAPH is an oxidizing agent. When an AAPH molecule is thermally broken down, C-radical molecules are produced. These molecules subsequently combine with oxygen to form peroxyl radical molecules^105^. In response to AAPH free radical-induced hemolysis, Sv-CollNPs demonstrated a 96.5 ± 1.2% anti-hemolytic effect, while L-ascorbic acid (vitamin C) exhibited 95.8 ± 0.9%, based on three independent replicates (*n* = 3). The difference between both treatments was statistically non-significant (*P* > 0.05), indicating comparable protective effects. The CollNPs generated from the CFS of *S. xinghaiensis* exhibited 96.1% anti-hemolythic efficacy^12^. The anti-hemolytic efficacy shown in Sv-CollNPs could arise from the antioxidation strength of the (OH) groups found in compounds found in phenol or (Ar-OH) compounds, as well as NH_2_ groups in aromatic amine compounds, as verified by FTIR. Aromatic amines have strong antioxidant properties; nevertheless, amino-substituted phenol is thought to be more potent than hydroxy-substituted ones^106^.

### *In vitro* cytotoxicity of Sv-CollNPs

**Fig. 12:**
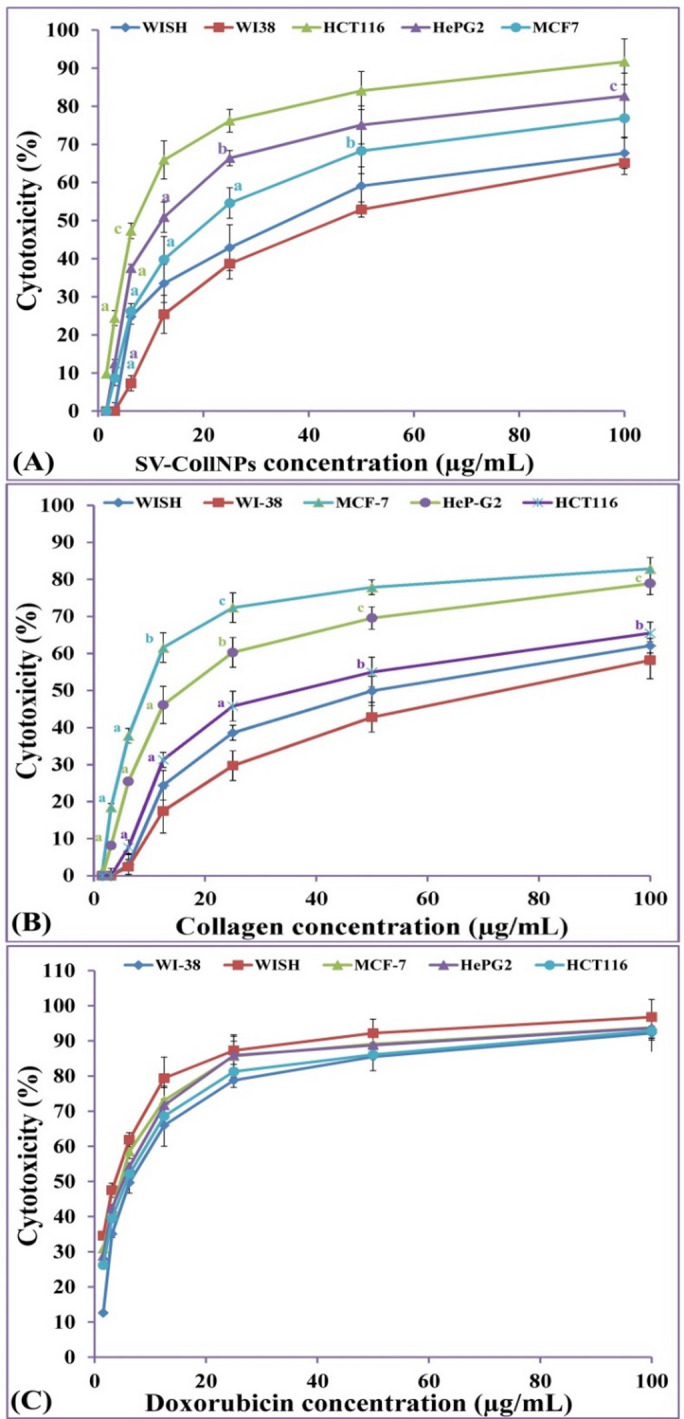
Cytotoxicity effects of (A) Sv-CollNPs, (B) collagen, and (C) DOX. Data are presented as mean ± SD (*n* = 3). Statistical significance compared with DOX was indicated as *P* < 0.05 (c), *P <* 0.01(b), and *P* ***<*** 0.001 (a). Both collagen and Sv-CollNPs showed a significant reduction in cytotoxicity toward normal cells compared to DOX (*P* < 0.001).

In this research, the in-vitro cytotoxicity of Sv-CollNPs, collagen, and DOX (Fig. 12A–C) have been studied against different cancer and normal cell lines using MTT analysis, which is considered one of the most common methods for determining the cytotoxic effect of different drugs with different doses. The MTT analysis is based on that mitochondrial activity in most living cells. Therefore, there is a clear correlation between variations in the number of living cells and alterations in their mitochondrial activities^107^. After 48 h of incubation, the inhibition activity became noticeable. Half-maximal inhibitory concentrations (IC_50_) values of cells after 48 h of incubation were calculated with the comparison of untreated cells. For Sv-CollNPs against cancer cell lines, the IC_50_ values were as follows: MCF-7 recorded 7.94 ± 0.6 µg/mL, HeP-G2 recorded 13.89 ± 1.0 µg/mL (strong inhibition), and HCT116 recorded 22.06 ± 1.6 µg/mL (moderate inhibition). Sv-CollNPs’ IC_50_ against normal cell lines, such as WI-38 and WISH, was determined to be 34.57 ± 2.2 and 45.68 ± 2.7 µg/mL (moderate inhibition), respectively (Table 6). Consequently, in contrast to normal cell lines (WISH and WI-38), the biosynthesized Sv-CollNPs exerted significantly greater cytotoxic impacts on cancer cell lines (MCF-7, HCT116, and HeP-G2). One-way ANOVA followed by Tukey’s test revealed that both collagen and Sv-CollNPs showed significantly lower cytotoxicity than DOX toward normal cells at all tested concentrations (*P* < 0.001), confirming their biosafety. For cancer cells, Sv-CollNPs exhibited significant cytotoxicity compared to DOX at concentrations of 25–3.125 µg/mL for MCF-7 (*P* < 0.001) and 12.5–3.125 µg/mL for HePG2 (*P* < 0.01–0.001), while no significant difference was observed for HCT116 at higher concentrations (50–100 µg/mL). These findings suggest that the cytotoxicity of Sv-CollNPs varies depending on the cell type, with MCF-7, HCT116, and HeP-G2 being more susceptible to the nanoparticles’ effects. This information is valuable for understanding the potential therapeutic or toxicological applications of Sv-CollNPs in targeting specific cell types.

According to Han et al.^108^, at a dosage of 0.2 mg/mL, marine collagen inhibited the development of HeLa and Hep-G2 cells by 38% and 50%, respectively. The IC_50_ of CollNPs biosynthesized using the CFS of *S. plicatus* against MCF-7 recorded 7.80 ± 0.5 µg/mL, HCT116 recorded 24.82 ± 1.7 µg/mL, and HeP-G2 recorded 13.36 ± 1.0 µg/mL that coordinate with our results^34^. Also, the IC_50_ of CollNPs biosynthesized using the CFS of *S. xinghaiensis* against MCF-7 was 11.62 ± 0.8 µg/mL, HeP-G2 was 19.60 ± 1.2 µg/mL, and HCT116 was 41.67 ± 2.2 µg/mL^12^.

Sv-CollNPs exhibited a greater cytotoxicity than the collagen standard. Many studies have indicated that the NPs exhibit greater toxicity than their larger counterparts, indicating that NPs are generally more effective in causing damage. This suggests that Sv-CollNPs may have cytotoxicity on enzymes found in the Golgi apparatus, lysosomal compartments, endoplasmic reticulum, and mitochondria, which convert MTT dye to purple formazan^12,109,110^.

MTT dye is converted to purple formazan when NADH, D-glucose, or other concentrations in the culture media are reduced. Therefore, CollNPs have a toxic impact on mitochondrial function^111^. According to El-Sawah et al.^12^, CollNPs have a toxic impact due to the substantial generation of peroxides, hydroxyl radical and reactive oxygen species.

**Table 6:** Demonstrating the growth inhibitory concentration (IC_50_) values of Sv-CollNPs against various normal and cancer cell lines, which were biosynthesized using *Streptomyces* sp. NEAA-5, collagen, and doxorubicin.

The cell lines	*In vitro* Cytotoxicity IC_50_ (µg/mL)		
	DOX	Collagen	Sv-CollNPs
WI-38	6.72 ± 0.5	66.79 ± 3.3^**^	34.57 ± 2.2^**^
WISH	3.34 ± 0.2	53.25 ± 2.8^**^	45.68 ± 2.7^**^
HCT116	5.23 ± 0.3	41.67 ± 2.2^***^	22.06 ± 1.6^***^
HeP-G2	4.50 ± 0.2	19.60 ± 1.2^**^	13.89 ± 1.0^**^
MCF-7	4.17 ± 0.2	11.62 ± 0.8^***^	7.94 ± 0.6^***^

Values are mean ± SD (*n* = 3). Significance vs. DOX: *P* < 0.05*, *P* < 0.01**, *P* < 0.001***.

### *In vivo* cytotoxicity of Sv-CollNPs

The scientific community is very interested in combined therapies for cancer treatment due to of their synergistic antitumor effects, decreasing the toxicity that is associated with individual drugs, and suppression of multidrug resistance through different modes of action^112^. Since collagen is the most abundant protein in many living organisms, it is utilized in tissue engineering and medical applications because it is an effective and safe biomaterial. Collagen-based nanoparticles have the ability to tolerate high temperatures, reduce a drug’s systemic toxicity, and enhance the cellular uptake of NPs owing to their biodegradability, biocompatibility, and low^34,113^.

**Fig. 13:**
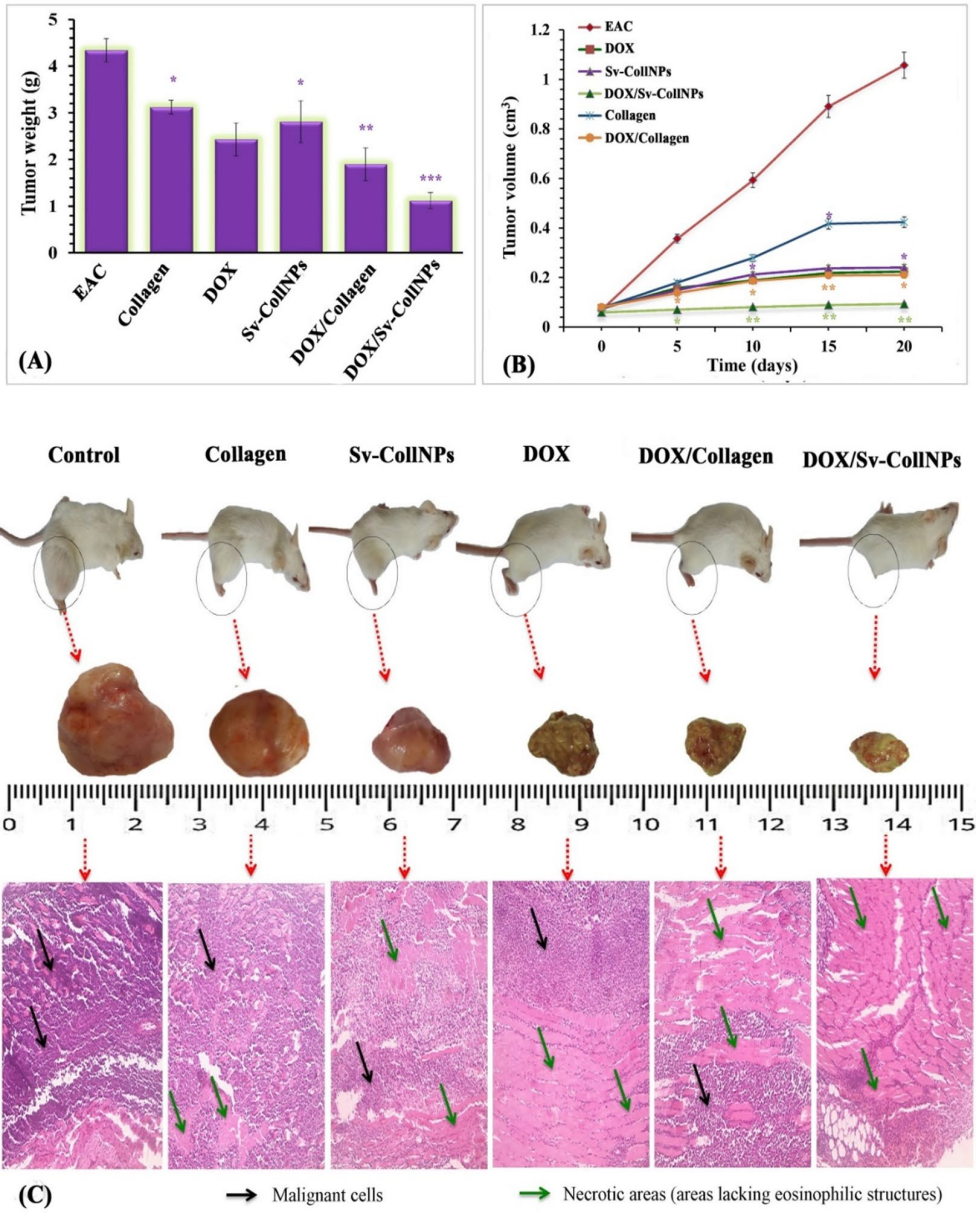
Suppression impacts of Sv-CollNPs, collagen, and their combination with DOX on tumor volume (A) and tumor weight (B) in EAC-bearing mice. Tumor volume progression is presented as mean ± SD (*n* = 8). Significant differences versus DOX at corresponding time points are indicated by asterisks (*P* < 0.05^*^, *P* < 0.01^**^, *P* < 0.01^***^). Shown are representative images of mice, excised tumors (captured under identical magnification, zoom, and camera distance), and (C) histopathological micrographs of tumor sections. Magnifications 100X. Scale bar 100 μm.

Sv-CollNPs and DOX/Sv-CollNPs in synergistic combination treatment were assessed for their synergistic efficacy in promoting apoptosis in EAC solid tumors (Fig. 13A–C; Table 7). After receiving treatment for 20 days, the mean tumor volume in the EAC control group rose from approximately 0.07 to 1.06 cm^3^. Administration of collagen, Sv-CollNPs, and DOX significantly reduces tumor growth in EAC-bearing mice by 65.66%, 83.84%, and 85.86%, respectively, when compared to EAC control mice. Furthermore, mice receiving a combination treatment of Sv-CollNPs/DOX (1.12 ± 0.35 g) demonstrated significant tumor growth suppression (97.98% inhibition) in comparison to mice receiving DOX (2.43 ± 0.31 g) and Sv-CollNPs (2.81 ± 0.26 g) alone. Statistical comparison with the DOX-treated group revealed time-dependent differences in tumor volume among treatments. Significant variations began to appear on day 5 and continued thereafter. Specifically, collagen + DOX showed a mild but significant reduction compared to DOX at day 5 (*P <* 0.05), while the combination of DOX and Sv-CollNPs demonstrated a stronger effect with significance maintained from day 5 to day 20 (*P* < 0.01). The statistical analysis at the end of the experiment confirmed these trends showed significant differences in tumor response among treatments (*P*-value = 0.00479). Collagen alone differed significantly from Sv-CollNPs (*P*-value = 0.0201), DOX/collagen (*P*-value = 0.0243), and DOX/Sv-CollNPs (*P*-value = 0.0037). This suggests that combination therapies, especially those with DOX and nanoparticles, may be more effective or have distinct effects compared to collagen alone.

The histopathological examination of the eosin and hematoxylin-stained tumor sections is presented in Fig. 13C. In the untreated mice (control), we observed that enormous malignant cells exhibiting condensed chromosomes proliferated rapidly, nuclear dyschromasia, anaplasia, numerous abnormal nuclei, and pleomorphism. Both DOX and Sv-CollNPs treatment of EAC mice resulted in slower proliferation rates in tumors, increased necrotic areas (areas without eosinophilic components), a significant increase in apoptotic cells, and mild inhibition. Giving DOX/Sv-CollNPs to mice with EAC resulted in a significant beneficial synergistic impact on the histopathological pattern. According to El-Sawah et al.^34^, the synergistic impact between DOX and CollNPs results in excessive ROS generation, which disrupts redox equilibrium in cancer cells. In general, nanoparticles have a stronger surface reactivity, owing to their nanoscale dimensions and elevated surface area, making them successful at causing apoptosis through ATP depletion, protein damage, lysosome disintegration, and glutathione depletion^114^. Collagen protein, a naturally occurring polymer, can liberate its constituent peptides by the action of the hydrolyzing enzymes. These peptides have a number of functions in the human body, including scavenging free radicals, reducing lipid oxidation, and aiding in maintaining the right free radicals’ balance^115^. When compared to normal cells, tumor cells have higher quantities of free radicals^116^. This means that Sv-CollNPs have the ability to slow down tumor growth of the EAC. The acute toxicity evaluation for marine-based collagen peptides revealed no significant adverse effect or safety concerns through the diet of 6658 mg/kg of body weight per day for males and 8586 mg/kg of body weight per day for females^117^. The LD_50_ of hydrolyzed marine collagen with a molecular weight of ~ 400, was found to exceed 2.5 g/kg body weight in Sprague Dawley CD rats, indicating that the type I marine collagen is completely safe^118^. Three female rats received oral administration of marine collagen with a dosage of 2 g/kg of their body weight, while an additional group of another 3 female rats were given the same dosage after fasting. Through the fourteen days that followed the dose, there were no recorded deaths or signs of systemic toxicity^118^.

**Table 7:** Suppression impact of Sv-CollNPs, collagen, DOX, DOX/collagen & DOX/Sv-CollNPs on tumor assessment parameters for EAC-bearing mice.

Groups	Parameters							
	Tumor wt. (g)	Tumor dimensions (cm)				Tumor volume average (cm^3^)		TGI (%)*
		0 day		20 day				
		L	S	L	S	0 day	20 day	
EAC	4.34 ± 0.4	0.61	0.47	1.53	1.18	0.07	1.06	0.0
Collagen	3.12 ± 0.55^*^	0.64	0.49	1.13	0.87	0.08	0.42	65.66
DOX	2.43 ± 0.31	0.61	0.47	0.9	0.69	0.07	0.21	85.86
Sv-CollNPs	2.81 ± 0.26^*^	0.64	0.47	0.98	0.7	0.08	0.24	83.84
DOX/collagen	1.9 ± 0.05^**^	0.65	0.49	0.89	0.69	0.08	0.21	86.87
DOX/Sv-CollNPs	1.12 ± 0.35^***^	0.65	0.5	0.74	0.53	0.08	0.1	97.98

*TGI: tumor growth inhibition. Values are mean ± SD (*n* = 8). Weight significance vs. DOX: *P* < 0.05*, *P* < 0.01**, *P* < 0.001***.

The collagen anticancer activity is largely attributed to its amino acid composition, which comprises histidine, serine, valine, leucine, phenylalanine, lysine, asparagine, glutamic acid, glycine, tyrosine, arginine, and hydroxyproline^34^. According to Yamaguchi et al.^119^, glutamic acid and aspartic acid could inhibit the growth of malignant cell development. Peptides that are high in arginine and lysine function as cationic molecules and can interact with the anionic membranes of cancer cells. This interaction may lead to membrane penetration, disruption of the cell membrane’s integrity, and potentially a contribution to the toxicity of the cancer cells^120^. Acidic environments cause histidine peptides to become protonated, which damages cancer cells via permeability of membrane^121^.

## Identification of *Streptomyces* strain NEAA-5

### Cultural and morphological characteristics

**Fig. 14:**
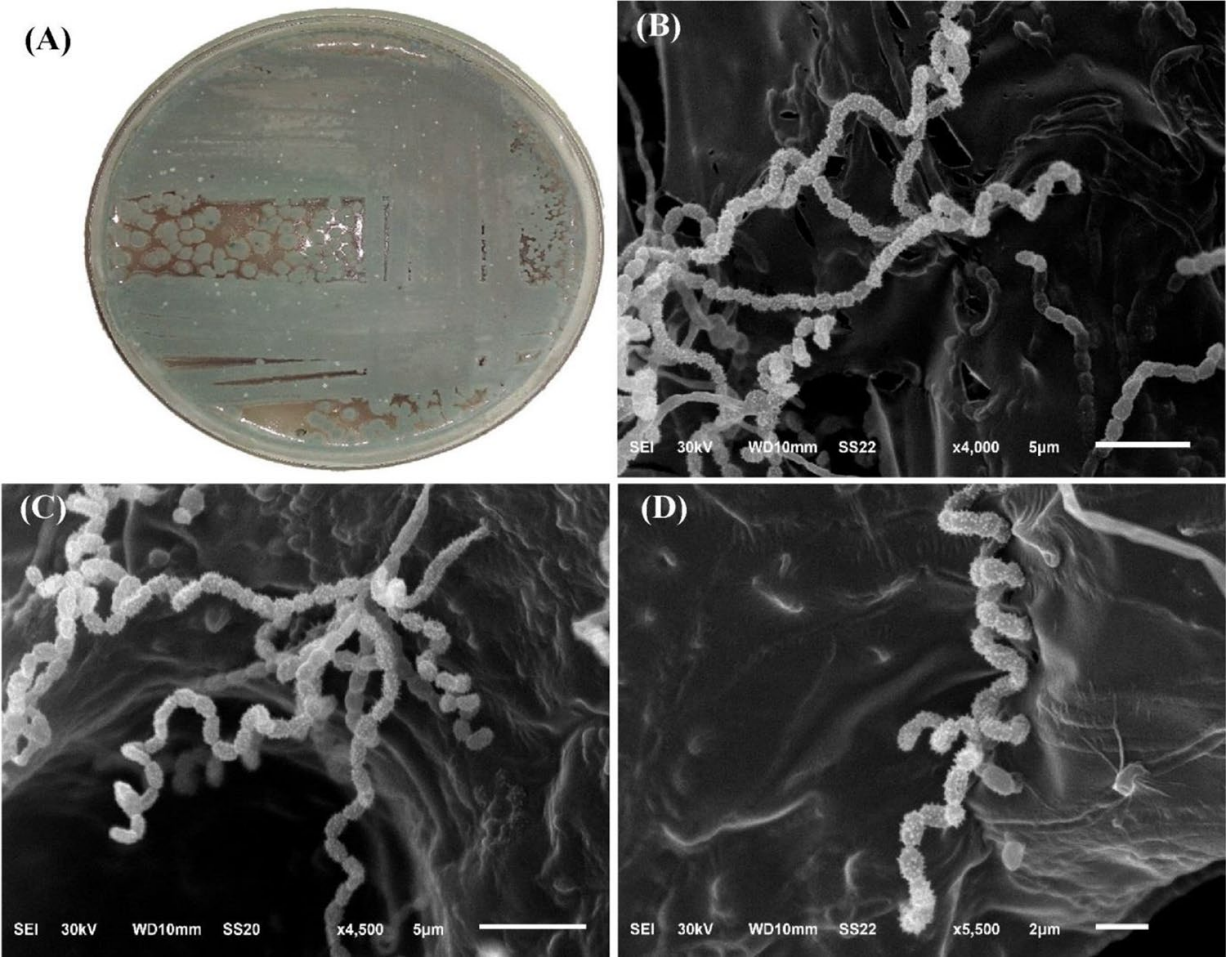
Growth of *Streptomyces* sp. strain NEAA-5 on starch-nitrate agar medium (A), and SEM images (B-D).

Morphological analysis of the 7–15-day-old *Streptomyces* strain NEAA-5 culture cultivated on Petri plates of starch nitrate media showed the abundance and good development of both substrate and aerial mycelium (Table 8; Fig. 14A). The morphological characteristics of *Streptomyces* strain NEAA-5 match those of the genus *Streptomyces*^122^. Mycelia of *Streptomyces* strain NEAA-5 were well developed on all tested media. The aerial mycelium was greenish grey in color, whereas the reverse surfaces were greenish black. Additionally, diffusible blue pigments are produced on all tested media.

SEM was utilized to investigate the morphology of *Streptomyces* strain NEAA-5 cultivated on yeast extract-malt extract agar (ISP2) at magnifications between 4000X and 5500X. This SEM micrograph demonstrates that there are no verticils or fragmentation of the mycelium present. *Streptomyces* strain NEAA-5 formed substrate mycelium and aerial hyphae that transformed into long spiral-shaped spore chains that could be either open or closed. Spore chains consist of elongated, cylindrical, non-motile spores with a spiny surface (Fig. 14B-D).

### Physiological characteristics

**Fig. 15:**
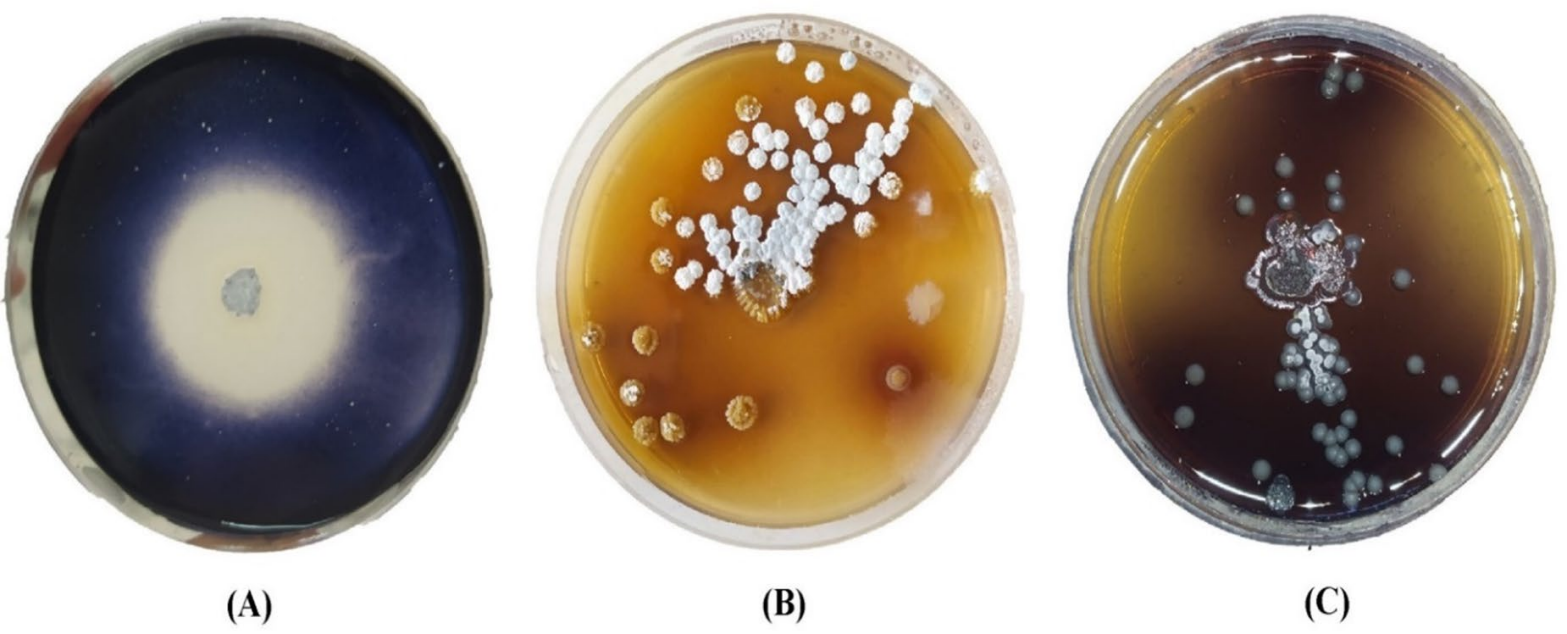
Starch hydrolysis by *Streptomyces* strain NEAA-5 (A), and Melanin pigment production (B-C).

*Streptomyces* strain NEAA-5’s physiological characteristics are illustrated in Table 8; Fig. 15. *Streptomyces* strain NEAA-5 *Streptomyces* strain NEAA-5 has the capacity to hydrolyze starch (Fig. 15A), coagulate milk, and liquefy gelatin. There is diffusible pigment (melanin) observed on peptone-yeast extract-iron agar and tryptone-yeast agar, and no diffusible pigment was noticed on glycerol-tyrosine agar (Fig. 15B, C). *Streptomyces* strain NEAA-5 showed a relatively high resistance to NaCl (7%, w/v). Maltose, lactose, and galactose were perfectly utilized as the sole carbon source. Xylose, glucose, and sucrose were moderately utilized as the sole carbon source. The growth of *Streptomyces* strain NEAA-5 occurred across pH values of 5–9 and temperatures of 25–40 °C, with maximum growth achieved at 30 °C and pH 7.

### Comparative 16 S rDNA gene analysis and phylogenetic analysis

**Fig. 16:**
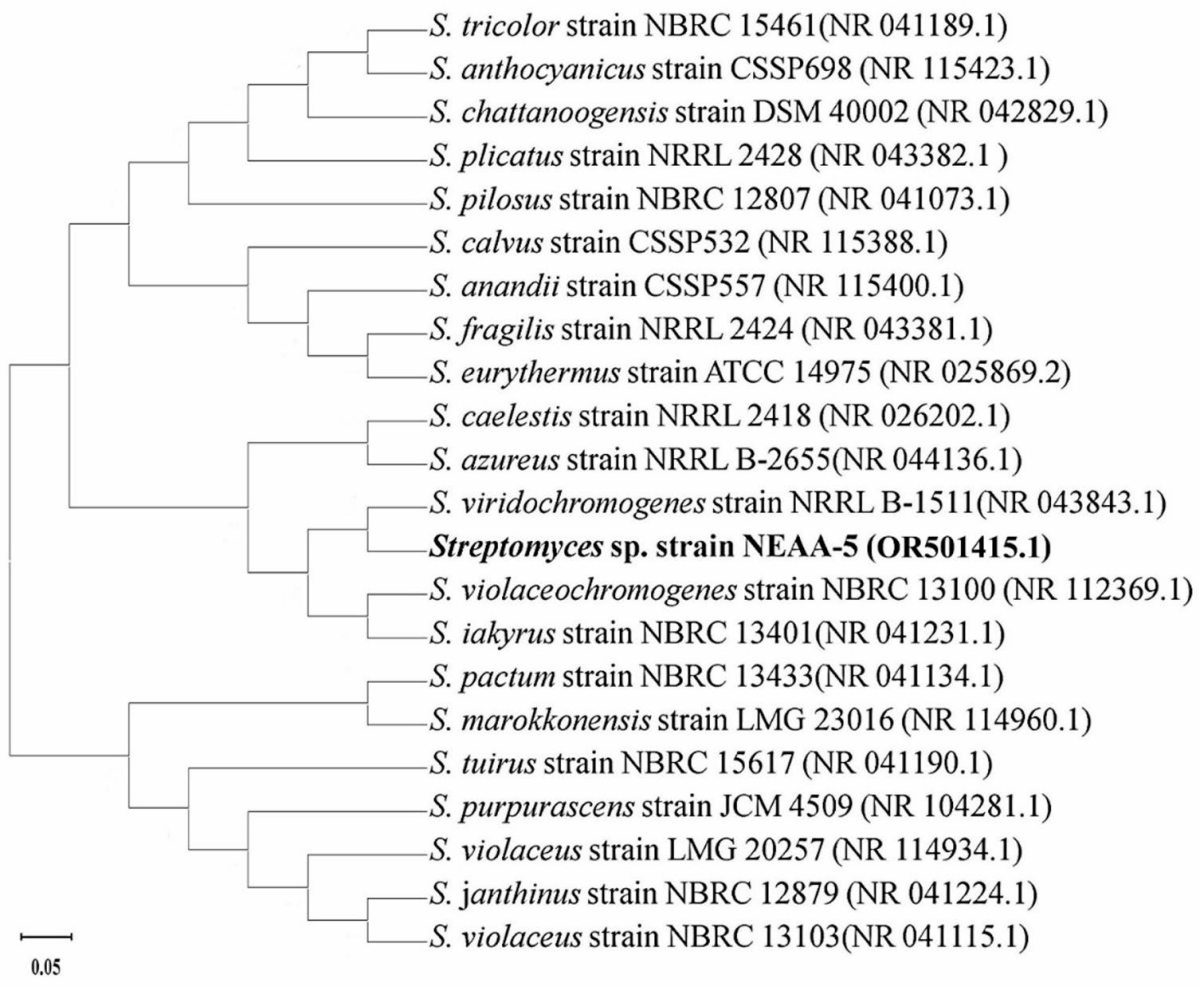
Neighbor-joining phylogenetic tree based on 16 S rDNA sequences.

The partial 16 S rDNA sequence of *Streptomyces* strain NEAA-5 was determined and deposited in the GenBank/EMBL/DDBJ database under the accession number OR501415.1. A BLAST search was performed to compare the sequence of strain NEAA-5 with those of closely related *Streptomyces* members. The results indicated a high level of similarity with several *Streptomyces* species. The phylogenetic tree (Fig. 16) was generated using the neighbor-joining method^123^ with the MEGA version 11 software package^44^. The analysis revealed that strain NEAA-5 clustered closely with several species within the genus *Streptomyces*, including *Streptomyces viridochromogenes* NRRL B-1511 (NR 043843.1; 98.42% similarity), *Streptomyces violaceochromogenes* NBRC 13,100 (NR 112369.1; 98.53%), *Streptomyces azureus* NRRL B-2655 (NR 044136.1; 98.18%), and *Streptomyces iakyrus* NBRC 13,401 (NR 041231.1; 98.53%).

**Table 8:** Phenotypic comparison of *Streptomyces* sp. strain NEAA-5 with closely related *Streptomyces* species.

Characteristic	*Streptomyces* sp. strain NEAA-5	*S. viridochromogenes*	*S. violaceochromogenes*
Aerial mycelium			
ISP medium 2	Greenish gray	Pale yellow green	Light grayish reddish brown
ISP medium 3	Greenish gray	Greenish gray or blue	Light grayish reddish brown
ISP medium 4	Greenish gray	Pale blue	Light grayish reddish brown
ISP medium 5	Greenish gray	Pale yellow green	Light grayish reddish brown
ISP medium 6	Greenish gray		
ISP medium 7	Greenish gray		
Reverse side of colony	Greenish black	Dark olive	Grayish yellow to strong brown
Spore chain morphology	Spirals	Spirals	Retinaculiaperti or Spirals
Spore surface	Spiny	Spiny	Smooth
Spore shape	Cylindrical with curved-surfaces	Cylindrical with curved-surfaces	
Glucose	+	+	+
Galactose	+++		
Fructose	˗	+	+
Xylose	+	+	+
Ribose	˗		
Sucrose	+	+	+
Lactose	+++		
Maltose	+++		
Maximum tolerance to NaCl conc. (% w/v)	7		
Melanin production	+	˗	+

+: Positive; −: Negative; starch hydrolysis, coagulation of milk, gelatin liquefaction were+; Blank cells: no data available.

Based on the phylogenetic tree and the comparison of morphological, cultural, and physiological characteristics with its closest relatives, strain NEAA-5 showed the highest similarity (98.42%) to *Streptomyces viridochromogenes* NRRL B-1511. Accordingly, based on its phenotypic characteristics and genetic similarity to *Streptomyces viridochromogenes*, the isolate NEAA-5 was designated as *Streptomyces viridochromogenes* NEAA-5.

## Conclusion

This study presents an innovative and eco-friendly approach for synthesizing Sv-CollNPs that exhibit diverse bioactive functionalities. In contrast to conventional chemical synthesis, that often rely on toxic reducing agents and energy-intensive processes, the biological process applied here utilizes metabolites from *Streptomyces viridochromogenes* strain NEAA-5, offering an eco-friendly, cost-effective, and biocompatible alternative. Moreover, this biosynthetic route also imparts intrinsic bioactive characteristics to the resulting nanoparticles, enhancing their potential for biomedical applications. Moreover, this biosynthetic route naturally incorporating bioactive compounds from the microbial metabolites into the structure of the nanoparticles, thereby enhancing their suitability for various biomedical applications. Comprehensive characterization confirmed the CFS as an effective biological crosslinking agent, facilitating the efficient transformation of collagen into Sv-CollNPs with a mean particle diameter of 30.41 nm. Process optimization markedly improved nanoparticles yield. Furthermore, both *in vitro* and *in vivo* studies demonstrated that the Sv-CollNPs are biocompatible and possess diverse functional properties, indicating their promise in biomedical applications. Future studies should focus on elucidating the mechanistic insights, large-scale production, and targeted drug delivery applications. Additionally, key challenges such as long-term stability, and in vivo biodistribution need to be addressed to fully realize their translational and clinical potential.

### Limitation

A limitation of the current study is that the stability of Sv-CollNPs in physiological media was not investigated. Although the nanoparticles were stored as a lyophilized powder providing good storage stability, their colloidal and biological stability upon re-dispersion should be evaluated in future studies. The precise mechanisms underlying the antioxidant and anticancer activities of Sv-CollNPs remain unclear; however, previous studies indicate that collagen-derived peptides and nanoparticle systems may exert their effects through modulation of ROS balance and mitochondrial integrity. Further mechanistic studies are required to validate these pathways. Future studies should explore systemic administration strategies and dose-dependent responses to improve the translational and therapeutic potential of Sv-CollNPs.

## Data Availability

All data generated or analyzed during this study are included in this article except the datasets that are available in the GenBank of The National Center for Biotechnology Information, (https://www.ncbi.nlm.nih.gov/nuccore/OR501412.1?report=GenBank).
